# An algorithm for automatic evaluation of the spot quality in two-color DNA microarray experiments

**DOI:** 10.1186/1471-2105-6-293

**Published:** 2005-12-09

**Authors:** Eugene Novikov, Emmanuel Barillot

**Affiliations:** 1 Service Bioinformatique, Institut Curie, 26 Rue d'Ulm, 75248 Paris Cedex 05, France.

## Abstract

**Background:**

Although DNA microarray technologies are very powerful for the simultaneous quantitative characterization of thousands of genes, the quality of the obtained experimental data is often far from ideal. The measured microarrays images represent a regular collection of spots, and the intensity of light at each spot is proportional to the DNA copy number or to the expression level of the gene whose DNA clone is spotted. Spot quality control is an essential part of microarray image analysis, which must be carried out at the level of individual spot identification. The problem is difficult to formalize due to the diversity of instrumental and biological factors that can influence the result.

**Results:**

For each spot we estimate the ratio of measured fluorescence intensities revealing differential gene expression or change in DNA copy numbers between the test and control samples. We also define a set of quality characteristics and a model for combining these characteristics into an overall spot quality value. We have developed a training procedure to evaluate the contribution of each individual characteristic in the overall quality. This procedure uses information available from replicated spots, located in the same array or over a set of replicated arrays. It is assumed that unspoiled replicated spots must have very close ratios, whereas poor spots yield greater diversity in the obtained ratio estimates.

**Conclusion:**

The developed procedure provides an automatic tool to quantify spot quality and to identify different types of spot deficiency occurring in DNA microarray technology. Quality values assigned to each spot can be used either to eliminate spots or to weight contribution of each ratio estimate in follow-up analysis procedures.

## Background

In comparative DNA microarray experiments compared test and control samples are labeled with different fluorescent dyes (typically the red-fluorescent Cy5 and the green-fluorescent Cy3), mixed up and co-hybridized with the DNA clones regularly spotted on the microarray. The array is scanned at a high spatial resolution at the corresponding fluorescent wavelengths, and the fluorescence intensities are recorded in two color channels (Cy5 and Cy3) for each pixel. The ratio of the measured intensities (Cy5/Cy3) for each microarray spot reveals either differential gene expression (cDNA technology [1]) or change in DNA copy numbers (comparative genome hybridization (CGH) technology [2]) between the test and control samples for the corresponding gene. Each ratio estimate should be accompanied by some measure of quality demonstrating the level confidence in the obtained ratios.

The main components of the microarray image analysis pipeline for spots include localization, quantification and quality control. Among these, quality control is the least formalized and least developed. To determine spot quality we need to have a clear definition of a good spot, or a list of all possible distortions that may spoil the spot. The diversity of instrumental platforms and instrumental and biological factors that may influence the result makes formalization difficult and unlikely to be universal.

In this paper, we consider the problem of quantifying spot quality in comparative DNA microarray experiments. Several attempts have been made to approach the problem [3-7]. Generally a number of parameters characterizing the spot, such as signal-to-noise ratio, size, circularity, etc., are introduced. These parameters have to be combined into an overall quality value to be used as a confidence level in the follow-up analysis. There are different methods for deriving such a parameter. For example, in two studies [5,6], it was assumed that individual quality scores contribute equivalently to the composite quality score. This may not be true, depending on the instrumental setup and experimental design. Therefore we need an approach that allows us to evaluate the weights that control the input of each of the marginal quality characteristics into the overall score. For that, different training procedures, in which the user classified a set of representative spots into three (accepted, rejected or intermediate spots) [3] or four (bad, close to bad, close to good or good spots) [7] groups, were proposed. This requires an expert to evaluate at least a couple of hundred spots to achieve a good approximation, which is a difficult and time-consuming task.

Here, we develop an automatic training procedure to evaluate the contribution (or weight) of each marginal quality characteristic into the overall quality score, together with an original set of quality characteristics and a model that maps this set into an overall quality value. This procedure is based on information from replicated spots, located on the same array or over a set of replicated arrays, and assumes that unspoiled replicated spots must have very close intensity ratios, whereas poor spots yield greater diversity in the obtained ratio estimates. The obtained weights can then be used to establish a critical limit for each quality characteristic, such that if a spot's characteristic exceeds its critical limit, the spot can be declared a "bad" spot.

We demonstrate the applicability of the developed algorithms using simulated artificial images and experimental images of different array designs used within our Institute and CGH images obtained from the UCSF Cancer Center.

## Results

We assume that the spots are identified and well localized [8], (Novikov E, Barillot E: unpublished data)] in squares (called spot cells). This involves: (i) identifying the position of each spot on the array to associate it with the spotted clone; and (ii) establishing the borders between the neighboring spots to allow further independent data processing (extracting quantitative information) for each spot. Visually this results in the generation of a grid covering the microarray image.

### Ratio estimation

We have implemented [8,9] two approaches for calculating the Cy5/Cy3 ratio for the spot: (i) based on spot segmentation and (ii) based on linear regression.

#### Spot segmentation

In the spot segmentation approach a direct arithmetic ratio of the background-corrected fluorescence intensity estimates in the two color channels is evaluated. This approach requires the identification of both the foreground – the measured spot – and the background – typically the level of non-specific hybridization. The segmentation procedure, in our implementation, is based on the *k*-means adaptive pixel-clustering algorithm [10] modified to explicitly take into consideration the geometrical constraints on the spots and to improve identification of the background areas for the spots with smooth edges.

#### Linear regression estimation

**Figure 1:**
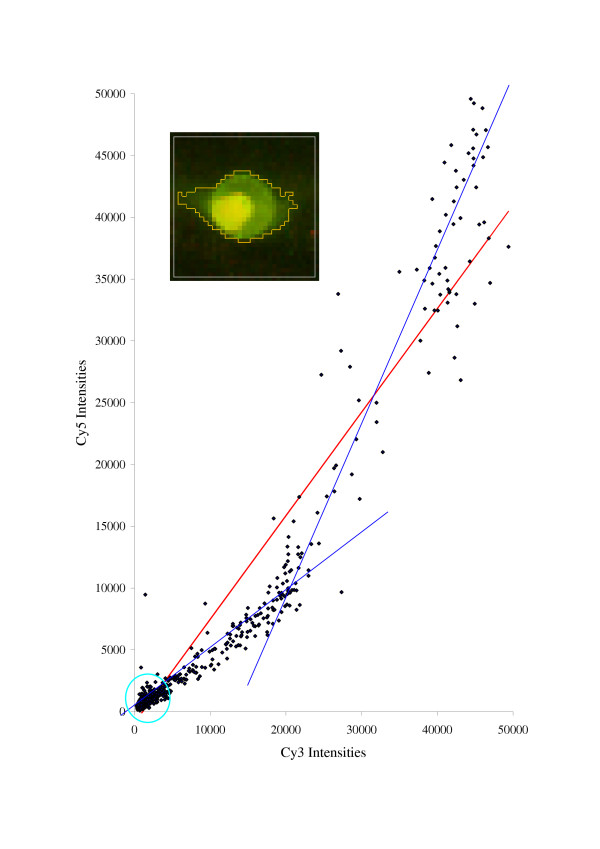
**Example of a spot with a low *DW *parameter (0.45). **Although the coefficient of determination of the linear regression plot (red line) is relatively high (0.92), it is obvious that the linear regression model is not appropriate in this case. It is possible that there are contributions (blue lines) from two different species occurring within the given spot, leading to two different Cy5/Cy3 ratios. The background pixels are grouped near the origin of the linear regression plot (cyan circle).

**Figure 2:**
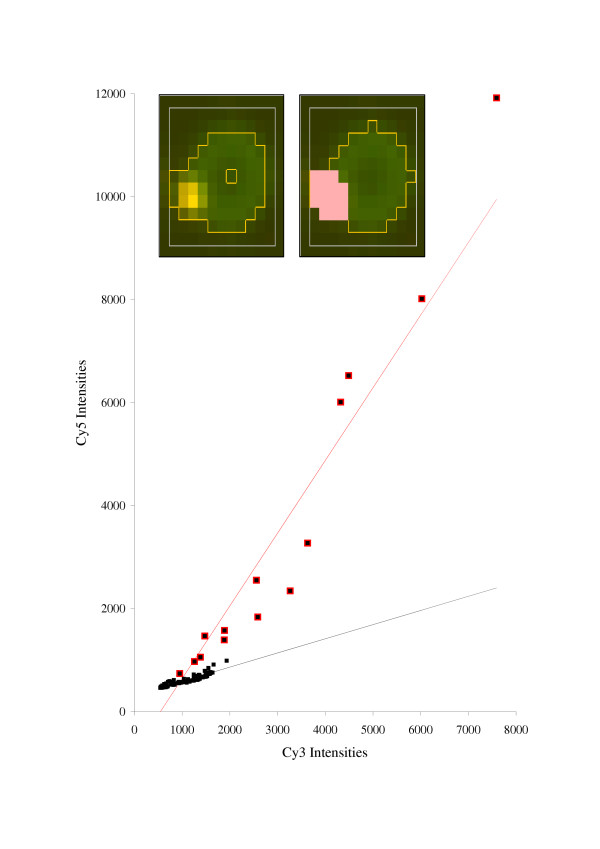
**Example of a spot with contamination. **The filtering procedure removes aberrant pixels (dots with the red contours), improving the Cy5/Cy3 ratio estimation and increasing the coefficient of determination of the linear regression plot. Before filtering (red line): *CD *= 0.86; linear regression ratio is 1.41; segmentation ratio is 0.712. After filtering (black line): *CD *= 0.91; linear regression ratio is 0.275; segmentation ratio is 0.273. However, larger amounts of aberrant pixels may result in a less reliable estimation.

In the linear regression approach, the ratio is represented as the slope of the linear regression fit of the pixel intensities in two color channels (Figs. 1 and 2). As measured fluorescence intensities are statistically distorted in both color channels, orthogonal regression [11,12] is used to estimate the slope. In this method spot segmentation is unnecessary, as background pixels are concentrated at the origin of the linear regression plot and do not influence the slope of the regression line (Fig. 1). However, outlier or aberrant pixels within the spot cells, even in small numbers, can strongly influence the regression line, thus biasing the ratio. We have improved [8,9] the linear regression approach by developing a statistical filtering procedure that systematically searches and removes aberrant or outlier pixels.

Briefly, this procedure can be outlined as follows. Suspicious pixels are examined by evaluating the quality of the linear regression fit with and without the suspicious pixel. We quantify the fit quality by the residual variance, *s*^2 ^[13]. The smaller *s*^2 ^is, the closer the linear regression line is to the experimental data. The ratio of the *s*^2 ^values is calculated for the fit with the tested pixel and for the fit without. If this ratio is larger than a critical value of the *F*-distribution at a user-defined confidence level, the pixel will be marked as aberrant. We select pixels with the highest intensity in either of two channels first and then select pixels having the largest deviation from the fitted regression line. To take into account the fact that the distortions caused by pixels from the top of the intensity scale and by pixels lying off of the linear regression line, may be different, we apply different confidence levels for the *F*-statistics for these pixels.

For the high-intensity pixels we also perform another test to determine how far their intensities are from the averaged intensity of the other pixels within the spot cell. This detects pixels, far away from the other pixels, that do not distort the linear regression line. Although these pixels may not change the Cy5/Cy3 ratio, they could be considered as aberrant pixels, as we expect to see an almost continuous distribution of pixels intensity.

The procedure performs iteratively until no more aberrant pixels are detected. An example of the outlier detection is presented in Fig. 2. Note that the regression approach is capable of detecting contamination pixels that are geometrically inseparable from the spot. Therefore, the developed procedure can be considered not only as a procedure for correcting ratio recovery, but also as a procedure to repair the spot and to improve the quality of experimental material. It requires, however, that the contamination clearly deviates from the straight regression line, which is defined by the majority of "good" pixels from the spot.

Although this procedure gives a high level of confidence in the linear regression ratio estimates, we still apply spot segmentation, because linear regression estimates may be biased when there is a high level of statistical noise (low correlation between the Cy3 and Cy5 color channels). However, after removing aberrant pixels, the segmentation algorithm also gives more robust estimates, and there is a greater agreement in the ratio values obtained with both methods.

As well as the Cy5/Cy3 ratio estimates, each of these approaches generates a series of parameters that can be used to evaluate the spot quality.

### Quality characteristics

We define a set of quality parameters, characterizing different features of the spot. These parameters are scaled between 0 (bad spot) and 1 (good spot) to facilitate further quality analysis.

First, we use the characteristics from the linear regression approach. The **coefficient of determination **(*CD *= *r*^2^, where *r *is the correlation coefficient) of linear regression signifies the degree of linear relationship between the intensities in the Cy3 and Cy5 channels. High values of *CD *(approaching 1) are expected for good spots. Low values suggest either relatively bright but non-correlated contamination, or strong statistical noise normally characterizing low-level (or missing) spots. Although the removal of aberrant pixels increases the *CD *of linear regression, it can still be low for noisy spots. These spots must be either flagged out or assigned a lower quality value. This parameter takes the range [0;1]: *q*_*1*_(*CD*) = *CD*.

The **Durbin-Watson statistic **(*DWS*) [14] evaluates the presence of the first-order autocorrelation in the residuals of the linear regression fit. It ranges from 0 to 4, 0 being a positive correlation and 4 being a negative correlation. A *DWS *value close to two indicates that the residuals are uncorrelated and the model is appropriate. Large deviations from two, resulting from systematic patterns in the residuals plot suggest that the spot cannot be modeled in terms of a simple linear regression. Low *DWS *values typically imply strong contamination that was not removed by filtering (Fig. 1). The Durbin-Watson quality parameter (*q*_2 _∈ [0;1]) is obtained from the *DWS *value by the following transformation: *q*_*2*_(*DWS*) = *1 *- |*DWS *- *2*|/*2*.

One more indicator of the quality directly available from the linear regression is the number (*N*) of the aberrant pixels flagged out by the filtering procedure. Although aberrant pixels can be found everywhere within the spot cell, we count only those pixels within the spot contours. This value can be used as a quantitative measure of **spot contamination**. Small numbers of aberrant pixels do not influence intensity ratio estimates, whereas the removal of large numbers of pixels from the spot may indicate inconsistency with the linear regression model (Fig. 2). We scale *N *to fit the range [0;1] as: *q*_*3*_(*N*) = *1 *- *N/S*, where *S *is the number of pixels within the spot contour.

We now define three quality parameters from the analysis of the contoured spot. The **diameter **of the spot is calculated as *D *= *2*(*S/π*)^*1/2*^. As the true value for the spot diameter may be difficult to establish, we use a typical value taken as the median diameter over all spots on the array. Spots with exceptionally small diameters should normally be penalized. We define the diameter quality parameter as *q*_*4*_(*D*) = *exp{D-T}*, if *D *<*T *and *q*_*4*_(*D*) = *1*, if *D *> *T*, where *T *is the typical diameter.

**Figure 3:**
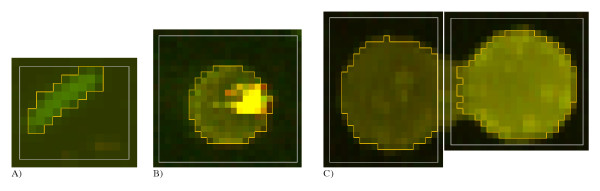
**Examples of spots with different types of distortions. **A) Large deviation from the circular shape (*GS *= 1.6); B) Bright piece of contamination within a larger circular spot, resulting in a low value of the *IS *parameter (*IS *= 1.71); C) Merged spots (*UB *= 2.98).

The **geometrical symmetry **parameter measures deviation of the contoured spot from the ideal circle. The center and the diameter of the ideal circle correspond to the center and the diameter of the real spot. We divide both the real spot and the ideal circle into eight segments (pie slices defined as [*kπ*/4;*(k *+ *1)π*/4], *k *= 0,...,7) and we count the number of pixels belonging to the spot (*N*_*si*_, *i *= 1,...,8) and to the circle (*N*_*ci*_, *i *= 1,...,8) for each segment. The sum of the absolute relative differences GS=∑i=18|Nsi−Nci|/Nci
 MathType@MTEF@5@5@+=feaafiart1ev1aaatCvAUfKttLearuWrP9MDH5MBPbIqV92AaeXatLxBI9gBaebbnrfifHhDYfgasaacH8akY=wiFfYdH8Gipec8Eeeu0xXdbba9frFj0=OqFfea0dXdd9vqai=hGuQ8kuc9pgc9s8qqaq=dirpe0xb9q8qiLsFr0=vr0=vr0dc8meaabaqaciaacaGaaeqabaqabeGadaaakeaacqWGhbWrcqWGtbWucqGH9aqpdaaeWbqaamaalyaabaWaaqWaaeaacqWGobGtdaqhaaWcbaGaem4CamNaemyAaKgabaaaaOGaeyOeI0IaemOta40aa0baaSqaaiabdogaJjabdMgaPbqaaaaaaOGaay5bSlaawIa7aaqaaiabd6eaonaaDaaaleaacqWGJbWycqWGPbqAaeaaaaaaaaqaaiabdMgaPjabg2da9iabigdaXaqaaiabiIda4aqdcqGHris5aaaa@46CB@ is then taken as an indicator of quality. For ideal circular spots *GS *should approach 0, whereas highly deformed (un-circular) spots can be recognized by high *GS *values (Fig. 3A). We transform the *GS *values as *q*_*5*_(*GS*) = *exp*(-*GS*) to fit the range [0;1].

Using the same partition of the spot into eight segments, we can also calculate the mean intensities for each of the segments (*I*_*i*_, *i *= 1,...,8). The **intensity symmetry **of the spot is defined as IS=∑i=18|Ii−I|/I
 MathType@MTEF@5@5@+=feaafiart1ev1aaatCvAUfKttLearuWrP9MDH5MBPbIqV92AaeXatLxBI9gBaebbnrfifHhDYfgasaacH8akY=wiFfYdH8Gipec8Eeeu0xXdbba9frFj0=OqFfea0dXdd9vqai=hGuQ8kuc9pgc9s8qqaq=dirpe0xb9q8qiLsFr0=vr0=vr0dc8meaabaqaciaacaGaaeqabaqabeGadaaakeaacqWGjbqscqWGtbWucqGH9aqpdaaeWbqaamaalyaabaWaaqWaaeaacqWGjbqsdaqhaaWcbaGaemyAaKgabaaaaOGaeyOeI0IaemysaKeacaGLhWUaayjcSdaabaGaemysaKeaaaWcbaGaemyAaKMaeyypa0JaeGymaedabaGaeGioaGdaniabggHiLdaaaa@3F95@, where *I *is the mean intensity within the spot. We scale the *IS *values in the range [0;1] as *q*_*6*_(*IS*) = *exp*(-*IS*). Although a spot may have perfect circular shape, it may contain very bright (or dark) and highly concentrated groups of pixels originating from pieces of dust or other contamination (Fig. 3B). *IS *is calculated for each of two channels (Cy3 and Cy5) and the maximal value is taken as a final estimate.

We estimate two Cy5/Cy3 ratios; one by the linear regression approach (*RR*), and the other by the segmentation algorithm (*RS*). Despite the different methods of estimation, the variation between the two obtained ratios should be as small as possible. Consistent results should be expected, as most of the contaminating pixels have been removed by the filtering procedure. Large variations between the two estimates may indicate a problematic spot. Therefore, we use the **coefficient of variation of two ratio estimates ***CVR *= *2*^*1/2*^|*RR-RS*|/(*RR*+*RS*) as a characteristic of quality. The *CVR *quality parameter is defined as *q*_*7*_(*CVR*) = *exp*(-*CVR*) fitting the range [0;1]. This measure of quality is the least explicit of all the quality characteristics: we can only determine that there is big discrepancy between the estimates but not why there is a big discrepancy, unless it is accompanied by lower values in any of the other quality parameters.

Finally, we introduce two parameters evaluating the quality of the background estimates. The first is the **uniformity of the background **(*UB*) around the spot, more precisely, along the grid lines separating neighborhood spots. We divide the grid line surrounding the spot into eight segments and calculate the mean intensity in each segment (*B*_*i*_, *i *= 1,...,8). The *UB *parameter is defined as: UB=∑i=18|Bi−B|/B
 MathType@MTEF@5@5@+=feaafiart1ev1aaatCvAUfKttLearuWrP9MDH5MBPbIqV92AaeXatLxBI9gBaebbnrfifHhDYfgasaacH8akY=wiFfYdH8Gipec8Eeeu0xXdbba9frFj0=OqFfea0dXdd9vqai=hGuQ8kuc9pgc9s8qqaq=dirpe0xb9q8qiLsFr0=vr0=vr0dc8meaabaqaciaacaGaaeqabaqabeGadaaakeaacqWGvbqvcqWGcbGqcqGH9aqpdaaeWbqaamaalyaabaWaaqWaaeaacqWGcbGqdaqhaaWcbaGaemyAaKgabaaaaOGaeyOeI0IaemOqaieacaGLhWUaayjcSdaabaGaemOqaieaaaWcbaGaemyAaKMaeyypa0JaeGymaedabaGaeGioaGdaniabggHiLdaaaa@3F61@, where *B *is the mean intensity for the whole grid line around the spot. Normally we would not expect to observe a big variability in the fluorescence intensity in the background areas. Large *UB *values may discover presence of relatively bright contamination around the spot, large variability in the background or merged neighboring spots (Fig. 3C). *UB *is rescaled to the range [0;1] by the exponential transformation: *q*_*8*_(*UB*) = *exp*(-*UB*).

The second background quality characteristic is the **absolute level of the background **(*AB*). We calculated this from the local area around the spot. As for the spot diameter *D*, there is no predefined ideal value for the absolute background. Therefore, as a benchmark for comparisons, we take the typical value as the median background level over all spots on the array. Spots with exceptionally high *AB *values should be treated with care or discarded. Of two background estimates obtained in two-color microarray experiments, we use one that gives the highest value as the most indicative of possible problems. We define the *AB *quality parameter as *q*_*9*_(*AB*) = *exp*{-(*AB*-*B*)/*B*}, if *AB *> *B *and *q*_*9*_(*AB*) = *1*, if *AB *<*B*, where *B *is the typical background level.

We cannot claim that the developed quality parameters are the optimal. However, they have led to reasonable results for most of the experimental and simulated situations we tested. Of course, there may be a possibility to formalize some of these parameters more precisely and/or to develop new parameters accounting for other types of distortions.

### Spot quality analysis

We consider two aims of spot quality analysis. The first is to combine the nine marginal quality parameters into an overall quality value. This value can be used either to flag out directly spots with a quality lower than a user-defined threshold, or, in the follow-up image analysis procedures (normalization, classification, clustering, etc.) as a parameter characterizing the level of confidence in the obtained Cy5/Cy3 ratios. The second aim is to identify a critical range (a sort of a confidence interval) for each quality characteristic. If a certain quality characteristic of the spot falls in this range, the corresponding spot could be classified as a "bad" spot.

#### Overall quality

**Figure 4:**
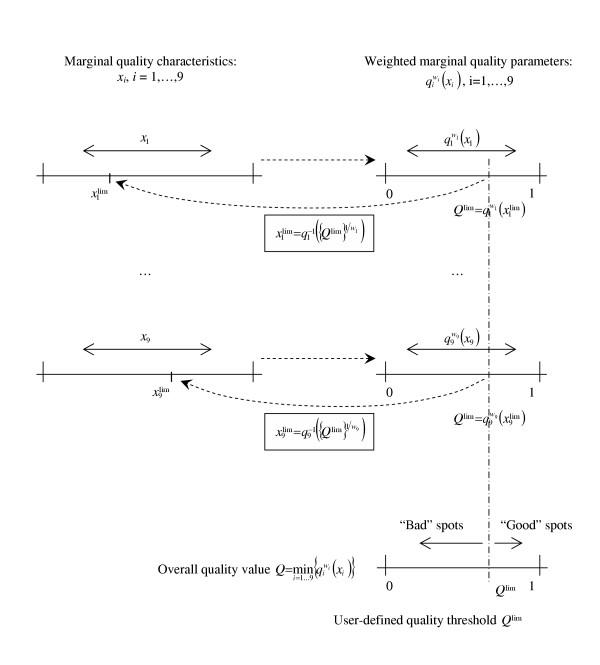
The correspondence between the quality characteristics, quality parameters and overall quality value.

We used the following definition for the overall quality value:

Q=min⁡i=1...9{qiwi},     (1)
 MathType@MTEF@5@5@+=feaafiart1ev1aaatCvAUfKttLearuWrP9MDH5MBPbIqV92AaeXatLxBI9gBaebbnrfifHhDYfgasaacH8akY=wiFfYdH8Gipec8Eeeu0xXdbba9frFj0=OqFfea0dXdd9vqai=hGuQ8kuc9pgc9s8qqaq=dirpe0xb9q8qiLsFr0=vr0=vr0dc8meaabaqaciaacaGaaeqabaqabeGadaaakeaacqWGrbqucqGH9aqpdaWfqaqaaiGbc2gaTjabcMgaPjabc6gaUbWcbaGaemyAaKMaeyypa0JaeGymaeJaeiOla4IaeiOla4IaeiOla4IaeGyoaKdabeaakmaacmaabaGaemyCae3aa0baaSqaaiabdMgaPbqaaiabdEha3naaDaaameaacqWGPbqAaeaaaaaaaaGccaGL7bGaayzFaaGaeiilaWIaaCzcaiaaxMaacqGGOaakcqaIXaqmcqGGPaqkaaa@4733@

where *q*_*i *_= *q*_*i*_(*x*_*i*_) ∈ [0;1], *i *= 1,...,9 are the marginal quality parameters for *x*_*1 *_= *CD*, *x*_*2 *_= *DWS*, *x*_*3 *_= *N*, *x*_*4 *_= *D*, *x*_*5 *_= *GS*, *x*_*6 *_= *IS*, *x*_*7 *_= *CVR*, *x*_*8 *_= *UB*, *x*_*9 *_= *AB*, and *w*_*i *_are the weights that control the input of the corresponding quality components into the overall quality value. A link between the weight *w*_*i *_and the critical value xilim⁡
 MathType@MTEF@5@5@+=feaafiart1ev1aaatCvAUfKttLearuWrP9MDH5MBPbIqV92AaeXatLxBI9gBaebbnrfifHhDYfgasaacH8akY=wiFfYdH8Gipec8Eeeu0xXdbba9frFj0=OqFfea0dXdd9vqai=hGuQ8kuc9pgc9s8qqaq=dirpe0xb9q8qiLsFr0=vr0=vr0dc8meaabaqaciaacaGaaeqabaqabeGadaaakeaacqWG4baEdaqhaaWcbaGaemyAaKgajeaybaGagiiBaWMaeiyAaKMaeiyBa0gaaaaa@3435@ can be established for each quality characteristic *i *= 1,...,9:

wi=log⁡{Qlim⁡}log⁡{qi(xilim⁡)},  or  xilim⁡=qi−1({Qlim⁡}1/wi),     (2)
 MathType@MTEF@5@5@+=feaafiart1ev1aaatCvAUfKttLearuWrP9MDH5MBPbIqV92AaeXatLxBI9gBaebbnrfifHhDYfgasaacH8akY=wiFfYdH8Gipec8Eeeu0xXdbba9frFj0=OqFfea0dXdd9vqai=hGuQ8kuc9pgc9s8qqaq=dirpe0xb9q8qiLsFr0=vr0=vr0dc8meaabaqaciaacaGaaeqabaqabeGadaaakeaacqWG3bWDdaqhaaWcbaGaemyAaKgabaaaaOGaeyypa0ZaaSaaaeaacyGGSbaBcqGGVbWBcqGGNbWzdaGadaqaaiabdgfarnaaDaaaleaaaeaacyGGSbaBcqGGPbqAcqGGTbqBaaaakiaawUhacaGL9baaaeaacyGGSbaBcqGGVbWBcqGGNbWzdaGadaqaaiabdghaXnaaDaaaleaacqWGPbqAaeaaaaGcdaqadaqaaiabdIha4naaDaaaleaacqWGPbqAaeaacyGGSbaBcqGGPbqAcqGGTbqBaaaakiaawIcacaGLPaaaaiaawUhacaGL9baaaaGaeiilaWIaaGPaVlaaykW7ieaacqWFVbWBcqWFYbGCcaaMc8UaaGPaVlabdIha4naaDaaaleaacqWGPbqAaeaacyGGSbaBcqGGPbqAcqGGTbqBaaGccqGH9aqpcqWGXbqCdaqhaaWcbaGaemyAaKgabaGaeyOeI0IaeGymaedaaOWaaeWaaeaadaGadaqaaiabdgfarnaaDaaaleaaaeaacyGGSbaBcqGGPbqAcqGGTbqBaaaakiaawUhacaGL9baadaqhaaWcbaaabaGaeGymaeJaei4la8Iaem4DaC3aa0baaWqaaiabdMgaPbqaaaaaaaaakiaawIcacaGLPaaacqGGSaalcaWLjaGaaCzcaiabcIcaOiabikdaYiabcMcaPaaa@7891@

where *Q*^lim ^∈ [0;1] is the user-defined overall quality threshold, and *q*_*i*_(xilim⁡
 MathType@MTEF@5@5@+=feaafiart1ev1aaatCvAUfKttLearuWrP9MDH5MBPbIqV92AaeXatLxBI9gBaebbnrfifHhDYfgasaacH8akY=wiFfYdH8Gipec8Eeeu0xXdbba9frFj0=OqFfea0dXdd9vqai=hGuQ8kuc9pgc9s8qqaq=dirpe0xb9q8qiLsFr0=vr0=vr0dc8meaabaqaciaacaGaaeqabaqabeGadaaakeaacqWG4baEdaqhaaWcbaGaemyAaKgajeaybaGagiiBaWMaeiyAaKMaeiyBa0gaaaaa@3435@) is the quality parameter calculated for xilim⁡
 MathType@MTEF@5@5@+=feaafiart1ev1aaatCvAUfKttLearuWrP9MDH5MBPbIqV92AaeXatLxBI9gBaebbnrfifHhDYfgasaacH8akY=wiFfYdH8Gipec8Eeeu0xXdbba9frFj0=OqFfea0dXdd9vqai=hGuQ8kuc9pgc9s8qqaq=dirpe0xb9q8qiLsFr0=vr0=vr0dc8meaabaqaciaacaGaaeqabaqabeGadaaakeaacqWG4baEdaqhaaWcbaGaemyAaKgajeaybaGagiiBaWMaeiyAaKMaeiyBa0gaaaaa@3435@. The critical value xilim⁡
 MathType@MTEF@5@5@+=feaafiart1ev1aaatCvAUfKttLearuWrP9MDH5MBPbIqV92AaeXatLxBI9gBaebbnrfifHhDYfgasaacH8akY=wiFfYdH8Gipec8Eeeu0xXdbba9frFj0=OqFfea0dXdd9vqai=hGuQ8kuc9pgc9s8qqaq=dirpe0xb9q8qiLsFr0=vr0=vr0dc8meaabaqaciaacaGaaeqabaqabeGadaaakeaacqWG4baEdaqhaaWcbaGaemyAaKgajeaybaGagiiBaWMaeiyAaKMaeiyBa0gaaaaa@3435@ sets up the limit such that if a certain characteristic *i *exceeds this limit, the corresponding quality parameter *q*_*i*_(xilim⁡
 MathType@MTEF@5@5@+=feaafiart1ev1aaatCvAUfKttLearuWrP9MDH5MBPbIqV92AaeXatLxBI9gBaebbnrfifHhDYfgasaacH8akY=wiFfYdH8Gipec8Eeeu0xXdbba9frFj0=OqFfea0dXdd9vqai=hGuQ8kuc9pgc9s8qqaq=dirpe0xb9q8qiLsFr0=vr0=vr0dc8meaabaqaciaacaGaaeqabaqabeGadaaakeaacqWG4baEdaqhaaWcbaGaemyAaKgajeaybaGagiiBaWMaeiyAaKMaeiyBa0gaaaaa@3435@) will become lower than *Q*^lim^. The correspondence between *x*_*i*_, xilim⁡
 MathType@MTEF@5@5@+=feaafiart1ev1aaatCvAUfKttLearuWrP9MDH5MBPbIqV92AaeXatLxBI9gBaebbnrfifHhDYfgasaacH8akY=wiFfYdH8Gipec8Eeeu0xXdbba9frFj0=OqFfea0dXdd9vqai=hGuQ8kuc9pgc9s8qqaq=dirpe0xb9q8qiLsFr0=vr0=vr0dc8meaabaqaciaacaGaaeqabaqabeGadaaakeaacqWG4baEdaqhaaWcbaGaemyAaKgajeaybaGagiiBaWMaeiyAaKMaeiyBa0gaaaaa@3435@, *q*_*i*_(*x*_*i*_), *q*_*i*_(xilim⁡
 MathType@MTEF@5@5@+=feaafiart1ev1aaatCvAUfKttLearuWrP9MDH5MBPbIqV92AaeXatLxBI9gBaebbnrfifHhDYfgasaacH8akY=wiFfYdH8Gipec8Eeeu0xXdbba9frFj0=OqFfea0dXdd9vqai=hGuQ8kuc9pgc9s8qqaq=dirpe0xb9q8qiLsFr0=vr0=vr0dc8meaabaqaciaacaGaaeqabaqabeGadaaakeaacqWG4baEdaqhaaWcbaGaemyAaKgajeaybaGagiiBaWMaeiyAaKMaeiyBa0gaaaaa@3435@), *w*_*i*_, *Q *and *Q*^lim ^is demonstrated in Fig. 4.

#### Quality weights *w*_*i*_

The experimental quality parameters *q*_*i*_, *i *= 1,...,9 are directly available from the quantification procedure, whereas the weights *w*_*i *_(or the critical values xilim⁡
 MathType@MTEF@5@5@+=feaafiart1ev1aaatCvAUfKttLearuWrP9MDH5MBPbIqV92AaeXatLxBI9gBaebbnrfifHhDYfgasaacH8akY=wiFfYdH8Gipec8Eeeu0xXdbba9frFj0=OqFfea0dXdd9vqai=hGuQ8kuc9pgc9s8qqaq=dirpe0xb9q8qiLsFr0=vr0=vr0dc8meaabaqaciaacaGaaeqabaqabeGadaaakeaacqWG4baEdaqhaaWcbaGaemyAaKgajeaybaGagiiBaWMaeiyAaKMaeiyBa0gaaaaa@3435@) are unknown and are not easily guessed or derived from theory. Therefore, the problem of spot quality analysis becomes a problem of weights (*w*_*i*_) estimation. This can only be solved if additional information is available. Here we consider three possibilities:

1. The additional information may come, for example, from the user expertise. The user has to classify the spots manually [3,7] and assign a quality value to each spot from a representative subset. These values are then used for training the model (1) leading to a combination of the weights (*w*_*i*_) such that the overall quality values reproduce the user classification reasonably well.

2. We can manually apply different combinations of the weights *w*_*i *_and visually appreciate, which spots have been flagged out. The trials must be continued until most of the user classified "bad" spots are eliminated by the chosen combinations of the weights.

3. The weights can be estimated automatically using information available from replicated spots on the same array or over a set of replicated arrays. Unspoiled replicate spots should have very similar estimated Cy5/Cy3 ratio values. Large differences between the observed ratios in the replicate spots would signal that some spots from this replicate were irregular. We formalize this approach by first defining the quality value for the replicate:

Qk=min⁡j=1...n{Qkj},     (3)
 MathType@MTEF@5@5@+=feaafiart1ev1aaatCvAUfKttLearuWrP9MDH5MBPbIqV92AaeXatLxBI9gBaebbnrfifHhDYfgasaacH8akY=wiFfYdH8Gipec8Eeeu0xXdbba9frFj0=OqFfea0dXdd9vqai=hGuQ8kuc9pgc9s8qqaq=dirpe0xb9q8qiLsFr0=vr0=vr0dc8meaabaqaciaacaGaaeqabaqabeGadaaakeaacqWGrbqudaqhaaWcbaGaem4AaSgabaaaaOGaeyypa0ZaaCbeaeaacyGGTbqBcqGGPbqAcqGGUbGBaSqaaiabdQgaQjabg2da9iabigdaXiabc6caUiabc6caUiabc6caUiabd6gaUbqabaGcdaGadaqaaiabdgfarnaaDaaaleaacqWGRbWAcqWGQbGAaeaaaaaakiaawUhacaGL9baacqGGSaalcaWLjaGaaCzcaiabcIcaOiabiodaZiabcMcaPaaa@4755@

where *k *indicates the replicates, *n *is the number of spots in a replicate and *Q*_*kj *_is a spot quality value given by Eq. (1). Substituting Eq. (1) into (3) yields

Qk=min⁡j=1...n{min⁡i=1...9{qkjiwi}},     (4)
 MathType@MTEF@5@5@+=feaafiart1ev1aaatCvAUfKttLearuWrP9MDH5MBPbIqV92AaeXatLxBI9gBaebbnrfifHhDYfgasaacH8akY=wiFfYdH8Gipec8Eeeu0xXdbba9frFj0=OqFfea0dXdd9vqai=hGuQ8kuc9pgc9s8qqaq=dirpe0xb9q8qiLsFr0=vr0=vr0dc8meaabaqaciaacaGaaeqabaqabeGadaaakeaacqWGrbqudaqhaaWcbaGaem4AaSgabaaaaOGaeyypa0ZaaCbeaeaacyGGTbqBcqGGPbqAcqGGUbGBaSqaaiabdQgaQjabg2da9iabigdaXiabc6caUiabc6caUiabc6caUiabd6gaUbqabaGcdaGadaqaamaaxababaGagiyBa0MaeiyAaKMaeiOBa4galeaacqWGPbqAcqGH9aqpcqaIXaqmcqGGUaGlcqGGUaGlcqGGUaGlcqaI5aqoaeqaaOWaaiWaaeaacqWGXbqCdaqhaaWcbaGaem4AaSMaemOAaOMaemyAaKgabaGaem4DaC3aaSbaaWqaaiabdMgaPbqabaaaaaGccaGL7bGaayzFaaaacaGL7bGaayzFaaGaeiilaWIaaCzcaiaaxMaacqGGOaakcqaI0aancqGGPaqkaaa@5984@

where *q*_*kji *_is the *i*-th quality parameter of the *j*-th replicated spot in the *k*-th replicate. The weights *w*_*i*_, *i *= 1,...,9 are the parameters that ensure the best fit of the experimental quality values (*Q*_*k *_*versus V*_*k*_) to a user-defined ideal quality curve *f*(*V*_*k*_), where *V*_*k *_is the intensity ratio variation coefficient in the *k*-th replicate. The best fit of *Q*_*k*_(*V*_*k*_) to *f*(*V*_*k*_) can be achieved by minimizing the sum of squared differences between *Q*_*k*_(*V*_*k*_) and *f*(*V*_*k*_) (least-squares fit):

∑kψk[f(Vk)−min⁡j=1...n{min⁡i=1...9{qkjiwi}}]2,     (5)
 MathType@MTEF@5@5@+=feaafiart1ev1aaatCvAUfKttLearuWrP9MDH5MBPbIqV92AaeXatLxBI9gBaebbnrfifHhDYfgasaacH8akY=wiFfYdH8Gipec8Eeeu0xXdbba9frFj0=OqFfea0dXdd9vqai=hGuQ8kuc9pgc9s8qqaq=dirpe0xb9q8qiLsFr0=vr0=vr0dc8meaabaqaciaacaGaaeqabaqabeGadaaakeaadaaeqbqaaiabeI8a5naaDaaaleaacqWGRbWAaeaaaaGcdaWadaqaaiabdAgaMjabcIcaOiabdAfawnaaDaaaleaacqWGRbWAaeaaaaGccqGGPaqkcqGHsisldaWfqaqaaiGbc2gaTjabcMgaPjabc6gaUbWcbaGaemOAaOMaeyypa0JaeGymaeJaeiOla4IaeiOla4IaeiOla4IaemOBa4gabeaakmaacmaabaWaaCbeaeaacyGGTbqBcqGGPbqAcqGGUbGBaSqaaiabdMgaPjabg2da9iabigdaXiabc6caUiabc6caUiabc6caUiabiMda5aqabaGcdaGadaqaaiabdghaXnaaDaaaleaacqWGRbWAcqWGQbGAcqWGPbqAaeaacqWG3bWDdaWgaaadbaGaemyAaKgabeaaaaaakiaawUhacaGL9baaaiaawUhacaGL9baaaiaawUfacaGLDbaadaqhaaWcbaaabaGaeGOmaidaaaqaaiabdUgaRbqab0GaeyyeIuoakiabcYcaSiaaxMaacaWLjaGaeiikaGIaeGynauJaeiykaKcaaa@6674@

where ψ_*k *_are the weights of the contribution of each replicate into the sum (5). The quality weights *w*_*i *_can be estimated by minimization of Eq (5) using one of the algorithms for non-linear fitting [15].

#### Ideal quality curve *f*(*V*_*k*_)

**Figure 5:**
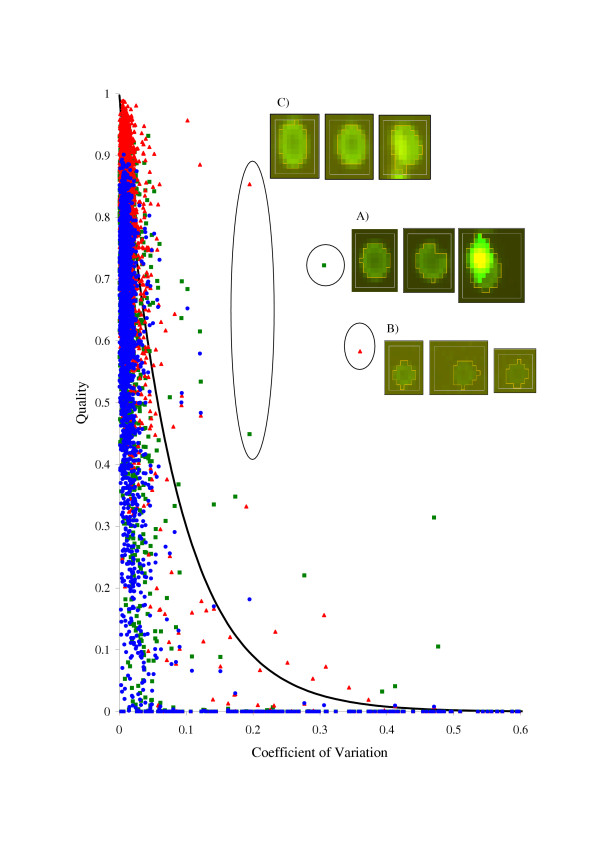
**Quality plots (*Q*_*k *_versus *V*_*k*_) for image 7A. ***Green dots *– using only the CD quality parameter; *red dots *– using only the *CRV *quality parameter; *blue dots *– using overall quality parameter *Q*. The black solid line is the exponential ideal quality curve *f*(*V*_*k*_) (Eq. (6),a). Three triplicates showing poor quality (outlined by circles) are given in the insets. The main characteristics of the spots from the selected triplicates are given in Table 3.

*f*(*V*_*k*_) defines how fast the overall quality of the replicates should decrease with increasing ratio variation. The shape of the ideal quality curve *f*(*V*_*k*_) is somewhat arbitrary; the only requirement is that it should demonstrate monotonic decay. Further formalization for *f*(*V*_*k*_) is hardly possible until more information regarding the ideal quality behavior becomes available. Therefore we have to look for empirical approaches to define *f*(*V*_*k*_). For example, *f*(*V*_*k*_) can be implemented as a non-parametric function, which can be constructed by the user through the properly designed user interface. *f*(*V*_*k*_) can also be represented as a function (such as *f*(*V*_*k*_) = Vkα
 MathType@MTEF@5@5@+=feaafiart1ev1aaatCvAUfKttLearuWrP9MDH5MBPbIqV92AaeXatLxBI9gBaebbnrfifHhDYfgasaacH8akY=wiFfYdH8Gipec8Eeeu0xXdbba9frFj0=OqFfea0dXdd9vqai=hGuQ8kuc9pgc9s8qqaq=dirpe0xb9q8qiLsFr0=vr0=vr0dc8meaabaqaciaacaGaaeqabaqabeGadaaakeaacqWGwbGvdaqhaaWcbaGaem4AaSgabaGaeqySdegaaaaa@310C@ exp{-β*V*_*k*_} with two adjustable parameters α and β), which can yield different shapes depending on the parameters values. Finally, a library of different functional shapes for *f*(*V*_*k*_) can be created. In our work we follow the third approach: three shapes were implemented for testing:

f(Vk)={exp⁡{−Vk/V¯}, (a)exp⁡{−Vk2/V¯2}, (b)1/{1+Vk/V¯}, (c)     (6)
 MathType@MTEF@5@5@+=feaafiart1ev1aaatCvAUfKttLearuWrP9MDH5MBPbIqV92AaeXatLxBI9gBaebbnrfifHhDYfgasaacH8akY=wiFfYdH8Gipec8Eeeu0xXdbba9frFj0=OqFfea0dXdd9vqai=hGuQ8kuc9pgc9s8qqaq=dirpe0xb9q8qiLsFr0=vr0=vr0dc8meaabaqaciaacaGaaeqabaqabeGadaaakeaacqWGMbGzcqGGOaakcqWGwbGvdaWgaaWcbaGaem4AaSgabeaakiabcMcaPiabg2da9maaceaabaqbaeqabmqaaaqaaiGbcwgaLjabcIha4jabcchaWnaacmaabaGaeyOeI0IaemOvay1aaSbaaSqaaiabdUgaRbqabaGccqGGVaWlcuWGwbGvgaqeaaGaay5Eaiaaw2haaiabcYcaSiabbccaGiabcIcaOiabdggaHjabcMcaPaqaaiGbcwgaLjabcIha4jabcchaWnaacmaabaGaeyOeI0IaemOvay1aa0baaSqaaiabdUgaRbqaaiabikdaYaaakiabc+caViqbdAfawzaaraWaaWbaaSqabeaacqaIYaGmaaaakiaawUhacaGL9baacqGGSaalcqqGGaaicqGGOaakcqWGIbGycqGGPaqkaeaadaWcgaqaaiabigdaXaqaamaacmaabaGaeGymaeJaey4kaSIaemOvay1aaSbaaSqaaiabdUgaRbqabaGccqGGVaWlcuWGwbGvgaqeaaGaay5Eaiaaw2haaiabcYcaSiabbccaGiabcIcaOiabdogaJjabcMcaPaaaaaaacaGL7baacaWLjaGaaCzcaiabcIcaOiabiAda2iabcMcaPaaa@6B4E@

and for each shape only the characteristic ratio variation coefficient V¯
 MathType@MTEF@5@5@+=feaafiart1ev1aaatCvAUfKttLearuWrP9MDH5MBPbIqV92AaeXatLxBI9gBaebbnrfifHhDYfgasaacH8akY=wiFfYdH8Gipec8Eeeu0xXdbba9frFj0=OqFfea0dXdd9vqai=hGuQ8kuc9pgc9s8qqaq=dirpe0xb9q8qiLsFr0=vr0=vr0dc8meaabaqaciaacaGaaeqabaqabeGadaaakeaacuWGwbGvgaqeaaaa@2DF9@ must be predefined. A typical example of the quality plot with the exponential *f*(*V*_*k*_) (Eq. (6),a) is shown in Fig. 5.

In our quality analysis algorithm, user participation is limited to the definition of the ideal quality curve shape *f*(*V*_*k*_). This is somewhat simpler than deciding on the quality of several hundred spots, which is used to teach the algorithm in the manual approach. However, as with other solutions, this algorithm requires representative images to train the model. It is impossible to evaluate confidently the weight of the contribution of the diameter quality parameter, for example, if all spots in the array have the same diameter. Therefore, a careful selection of training images containing a realistic diversity of all possible distortions and artifacts is needed.

#### Fitting weights ψ_*k*_

An important issue of the minimization of Eq (5) is reasonable selection of the fitting weights ψ_*k*_. The easiest way is to set all ψ_*k *_equal to one. However, most of the replicates are often observed in the initial part of the quality plot (high-quality spots), whereas there may be only a few replicates in the tail of the plot (poor-quality spots). In this case, equalized weights would give a very accurate fit for the initial part of the quality curve while ignoring the tail. However, mainly the tail contains the relevant information (spots with different distortions and artifacts) for identifying the quality weights. Therefore, we try to increase the input from regions with a smaller number of replicates by defining the weights ψ_*k *_in the following way:

1. All replicates are sorted according to their ratio variation *V*_*k*_.

2. The set of sorted replicates is divided into bins with ten replicates in each bin. The difference *λ*_*j *_between the smallest and the largest ratio variation coefficients in each bin *j *is calculated. The larger this difference, the smaller the concentration of the replicates in the given region of the quality plot.

3. The weights ψ_*k *_for Eq. (5) are then calculated as ψ_*k *_= ψ_j*10+*i *_= λj0.5
 MathType@MTEF@5@5@+=feaafiart1ev1aaatCvAUfKttLearuWrP9MDH5MBPbIqV92AaeXatLxBI9gBaebbnrfifHhDYfgasaacH8akY=wiFfYdH8Gipec8Eeeu0xXdbba9frFj0=OqFfea0dXdd9vqai=hGuQ8kuc9pgc9s8qqaq=dirpe0xb9q8qiLsFr0=vr0=vr0dc8meaabaqaciaacaGaaeqabaqabeGadaaakeaacqaH7oaBdaqhaaWcbaGaemOAaOgabaGaeGimaaJaeiOla4IaeGynaudaaaaa@32B4@, *i *= 0,...,9. That is, they are equalized for ten replicates from the same bin *j*.

#### Follow-up image analysis

As it was mentioned earlier, the overall quality value, *Q *(Eq. (1)), can be used as a parameter characterizing the level of confidence in the obtained Cy5/Cy3 ratios. If, for example, *n *ratios should be averaged, the weighted mean would ensure a more robust estimate for the average:

R¯=∑l=1nQlRl∑l=1nQl,     (7)
 MathType@MTEF@5@5@+=feaafiart1ev1aaatCvAUfKttLearuWrP9MDH5MBPbIqV92AaeXatLxBI9gBaebbnrfifHhDYfgasaacH8akY=wiFfYdH8Gipec8Eeeu0xXdbba9frFj0=OqFfea0dXdd9vqai=hGuQ8kuc9pgc9s8qqaq=dirpe0xb9q8qiLsFr0=vr0=vr0dc8meaabaqaciaacaGaaeqabaqabeGadaaakeaacuWGsbGugaqeaiabg2da9maalaaabaWaaabCaeaacqWGrbqudaqhaaWcbaGaemiBaWgabaaaaOGaemOuai1aa0baaSqaaiabdYgaSbqaaaaaaeaacqWGSbaBcqGH9aqpcqaIXaqmaeaacqWGUbGBa0GaeyyeIuoaaOqaamaaqahabaGaemyuae1aa0baaSqaaiabdYgaSbqaaaaaaeaacqWGSbaBcqGH9aqpcqaIXaqmaeaacqWGUbGBa0GaeyyeIuoaaaGccqGGSaalcaWLjaGaaCzcaiabcIcaOiabiEda3iabcMcaPaaa@4A0A@

where *R*_*l *_is the Cy5/Cy3 ratio and *Q*_*l*_, is the corresponding overall quality value (*l *= 1,...,*n*). The weighted coefficient of variation is defined as

V=∑l=1nQl(Rl−R¯)2/∑l=1nQlR¯.     (8)
 MathType@MTEF@5@5@+=feaafiart1ev1aaatCvAUfKttLearuWrP9MDH5MBPbIqV92AaeXatLxBI9gBaebbnrfifHhDYfgasaacH8akY=wiFfYdH8Gipec8Eeeu0xXdbba9frFj0=OqFfea0dXdd9vqai=hGuQ8kuc9pgc9s8qqaq=dirpe0xb9q8qiLsFr0=vr0=vr0dc8meaabaqaciaacaGaaeqabaqabeGadaaakeaacqWGwbGvcqGH9aqpdaWcaaqaamaakaaabaWaaSGbaeaadaaeWbqaaiabdgfarnaaDaaaleaacqWGSbaBaeaaaaGcdaqadaqaaiabdkfasnaaDaaaleaacqWGSbaBaeaaaaGccqGHsislcuWGsbGugaqeaaGaayjkaiaawMcaamaaCaaaleqabaGaeGOmaidaaaqaaiabdYgaSjabg2da9iabigdaXaqaaiabd6gaUbqdcqGHris5aaGcbaWaaabCaeaacqWGrbqudaqhaaWcbaGaemiBaWgabaaaaaqaaiabdYgaSjabg2da9iabigdaXaqaaiabd6gaUbqdcqGHris5aaaaaSqabaaakeaacuWGsbGugaqeaaaacqGGUaGlcaWLjaGaaCzcaiabcIcaOiabiIda4iabcMcaPaaa@505A@

Note that the ratio variation coefficient *V*_*k *_from Eq. (5) can be determined from Eq. (8), if we set *Q*_*l *_= 1, *l *= 1,...,*n*, with *n *being the number of spots in the *k*-th replicate.

### Image simulation

In [9] we have described the Monte-Carlo simulation model for generating artificial microarray images. The advantage of using artificial images is that we always know the exact Cy5/Cy3 ratio values. This allows us to test and compare objectively different image analysis algorithms. The general model for the two-color (Cy3, Cy5) microarray image is given by [9]:

FCy3(x,y)=∑k=1Ng(x,y,cksx,cksy,rs,Is)+∑k=1Mg(x,y,ckdx,ckdy,rd,Id),     (9)
 MathType@MTEF@5@5@+=feaafiart1ev1aaatCvAUfKttLearuWrP9MDH5MBPbIqV92AaeXatLxBI9gBaebbnrfifHhDYfgasaacH8akY=wiFfYdH8Gipec8Eeeu0xXdbba9frFj0=OqFfea0dXdd9vqai=hGuQ8kuc9pgc9s8qqaq=dirpe0xb9q8qiLsFr0=vr0=vr0dc8meaabaqaciaacaGaaeqabaqabeGadaaakeaacqWGgbGrdaqhaaWcbaGaem4qamKaemyEaKNaeG4mamdabaaaaOGaeiikaGIaemiEaGNaeiilaWIaemyEaKNaeiykaKIaeyypa0ZaaabCaeaacqWGNbWzdaqadaqaaiabdIha4jabcYcaSiabdMha5jabcYcaSiabdogaJnaaDaaaleaacqWGRbWAaeaacqWGZbWCcqWG4baEaaGccqGGSaalcqWGJbWydaqhaaWcbaGaem4AaSgabaGaem4CamNaemyEaKhaaOGaeiilaWIaemOCai3aa0baaSqaaaqaaiabdohaZbaakiabcYcaSiabdMeajnaaDaaaleaaaeaacqWGZbWCaaaakiaawIcacaGLPaaaaSqaaiabdUgaRjabg2da9iabigdaXaqaaiabd6eaobqdcqGHris5aOGaey4kaSYaaabCaeaacqWGNbWzdaqadaqaaiabdIha4jabcYcaSiabdMha5jabcYcaSiabdogaJnaaDaaaleaacqWGRbWAaeaacqWGKbazcqWG4baEaaGccqGGSaalcqWGJbWydaqhaaWcbaGaem4AaSgabaGaemizaqMaemyEaKhaaOGaeiilaWIaemOCai3aa0baaSqaaaqaaiabdsgaKbaakiabcYcaSiabdMeajnaaDaaaleaaaeaacqWGKbazaaaakiaawIcacaGLPaaaaSqaaiabdUgaRjabg2da9iabigdaXaqaaiabd2eanbqdcqGHris5aOGaeiilaWIaaCzcaiaaxMaacqGGOaakcqaI5aqocqGGPaqkaaa@8214@

FCy5(x,y)=R∑k=1Ng(x,y,cksx,cksy,rs,Is)+∑k=1Mg(x,y,ckdx,ckdy,rd,Id),     (10)
 MathType@MTEF@5@5@+=feaafiart1ev1aaatCvAUfKttLearuWrP9MDH5MBPbIqV92AaeXatLxBI9gBaebbnrfifHhDYfgasaacH8akY=wiFfYdH8Gipec8Eeeu0xXdbba9frFj0=OqFfea0dXdd9vqai=hGuQ8kuc9pgc9s8qqaq=dirpe0xb9q8qiLsFr0=vr0=vr0dc8meaabaqaciaacaGaaeqabaqabeGadaaakeaacqWGgbGrdaqhaaWcbaGaem4qamKaemyEaKNaeGynaudabaaaaOGaeiikaGIaemiEaGNaeiilaWIaemyEaKNaeiykaKIaeyypa0JaemOuai1aaabCaeaacqWGNbWzdaqadaqaaiabdIha4jabcYcaSiabdMha5jabcYcaSiabdogaJnaaDaaaleaacqWGRbWAaeaacqWGZbWCcqWG4baEaaGccqGGSaalcqWGJbWydaqhaaWcbaGaem4AaSgabaGaem4CamNaemyEaKhaaOGaeiilaWIaemOCai3aa0baaSqaaaqaaiabdohaZbaakiabcYcaSiabdMeajnaaDaaaleaaaeaacqWGZbWCaaaakiaawIcacaGLPaaaaSqaaiabdUgaRjabg2da9iabigdaXaqaaiabd6eaobqdcqGHris5aOGaey4kaSYaaabCaeaacqWGNbWzdaqadaqaaiabdIha4jabcYcaSiabdMha5jabcYcaSiabdogaJnaaDaaaleaacqWGRbWAaeaacqWGKbazcqWG4baEaaGccqGGSaalcqWGJbWydaqhaaWcbaGaem4AaSgabaGaemizaqMaemyEaKhaaOGaeiilaWIaemOCai3aa0baaSqaaaqaaiabdsgaKbaakiabcYcaSiabdMeajnaaDaaaleaaaeaacqWGKbazaaaakiaawIcacaGLPaaaaSqaaiabdUgaRjabg2da9iabigdaXaqaaiabd2eanbqdcqGHris5aOGaeiilaWIaaCzcaiaaxMaacqGGOaakcqaIXaqmcqaIWaamcqGGPaqkaaa@8423@

where *N *is the number of spots and *M *is the number of dust clusters, cksx
 MathType@MTEF@5@5@+=feaafiart1ev1aaatCvAUfKttLearuWrP9MDH5MBPbIqV92AaeXatLxBI9gBaebbnrfifHhDYfgasaacH8akY=wiFfYdH8Gipec8Eeeu0xXdbba9frFj0=OqFfea0dXdd9vqai=hGuQ8kuc9pgc9s8qqaq=dirpe0xb9q8qiLsFr0=vr0=vr0dc8meaabaqaciaacaGaaeqabaqabeGadaaakeaacqWGJbWydaqhaaWcbaGaem4AaSgabaGaem4CamNaemiEaGhaaaaa@326F@ and cksy
 MathType@MTEF@5@5@+=feaafiart1ev1aaatCvAUfKttLearuWrP9MDH5MBPbIqV92AaeXatLxBI9gBaebbnrfifHhDYfgasaacH8akY=wiFfYdH8Gipec8Eeeu0xXdbba9frFj0=OqFfea0dXdd9vqai=hGuQ8kuc9pgc9s8qqaq=dirpe0xb9q8qiLsFr0=vr0=vr0dc8meaabaqaciaacaGaaeqabaqabeGadaaakeaacqWGJbWydaqhaaWcbaGaem4AaSgabaGaem4CamNaemyEaKhaaaaa@3271@ are the coordinates of the center of a spot, ckdx
 MathType@MTEF@5@5@+=feaafiart1ev1aaatCvAUfKttLearuWrP9MDH5MBPbIqV92AaeXatLxBI9gBaebbnrfifHhDYfgasaacH8akY=wiFfYdH8Gipec8Eeeu0xXdbba9frFj0=OqFfea0dXdd9vqai=hGuQ8kuc9pgc9s8qqaq=dirpe0xb9q8qiLsFr0=vr0=vr0dc8meaabaqaciaacaGaaeqabaqabeGadaaakeaacqWGJbWydaqhaaWcbaGaem4AaSgabaGaemizaqMaemiEaGhaaaaa@3251@ and ckdy
 MathType@MTEF@5@5@+=feaafiart1ev1aaatCvAUfKttLearuWrP9MDH5MBPbIqV92AaeXatLxBI9gBaebbnrfifHhDYfgasaacH8akY=wiFfYdH8Gipec8Eeeu0xXdbba9frFj0=OqFfea0dXdd9vqai=hGuQ8kuc9pgc9s8qqaq=dirpe0xb9q8qiLsFr0=vr0=vr0dc8meaabaqaciaacaGaaeqabaqabeGadaaakeaacqWGJbWydaqhaaWcbaGaem4AaSgabaGaemizaqMaemyEaKhaaaaa@3253@ are the coordinates of the center of a dust cluster, *r*^*s *^and *r*^*d *^are the approximate radiuses of the spot and dust cluster, respectively, *I*^*s *^and *I*^*d *^are the fluorescence intensity in the center of the spot in the Cy3 color channel and in the center of the dust cluster, respectively, and *R *is the ratio of the test and control samples for each spot. Dust is represented by the random distribution over the array of clusters of pixels of varying brightness. We consider that these pixel clusters have an identical shape to the spots and therefore the same analytical representation is used for an ideal spot shape and dust cluster:

g(x,y,cx,cy,r,I)=Iexp⁡(−{[x−cxr]4+[y−cyr]4+[x−cxr]2[y−cyr]2}/2).     (11)
 MathType@MTEF@5@5@+=feaafiart1ev1aaatCvAUfKttLearuWrP9MDH5MBPbIqV92AaeXatLxBI9gBaebbnrfifHhDYfgasaacH8akY=wiFfYdH8Gipec8Eeeu0xXdbba9frFj0=OqFfea0dXdd9vqai=hGuQ8kuc9pgc9s8qqaq=dirpe0xb9q8qiLsFr0=vr0=vr0dc8meaabaqaciaacaGaaeqabaqabeGadaaakeaacqWGNbWzcqGGOaakcqWG4baEcqGGSaalcqWG5bqEcqGGSaalcqWGJbWydaqhaaWcbaaabaGaemiEaGhaaOGaeiilaWIaem4yam2aa0baaSqaaaqaaiabdMha5baakiabcYcaSiabdkhaYjabcYcaSiabdMeajjabcMcaPiabg2da9iabdMeajjGbcwgaLjabcIha4jabcchaWnaabmaabaGaeyOeI0YaaSGbaeaadaGadaqaamaadmaabaWaaSaaaeaacqWG4baEcqGHsislcqWGJbWydaqhaaWcbaaabaGaemiEaGhaaaGcbaGaemOCaihaaaGaay5waiaaw2faamaaDaaaleaaaeaacqaI0aanaaGccqGHRaWkdaWadaqaamaalaaabaGaemyEaKNaeyOeI0Iaem4yam2aa0baaSqaaaqaaiabdMha5baaaOqaaiabdkhaYbaaaiaawUfacaGLDbaadaqhaaWcbaaabaGaeGinaqdaaOGaey4kaSYaamWaaeaadaWcaaqaaiabdIha4jabgkHiTiabdogaJnaaDaaaleaaaeaacqWG4baEaaaakeaacqWGYbGCaaaacaGLBbGaayzxaaWaa0baaSqaaaqaaiabikdaYaaakmaadmaabaWaaSaaaeaacqWG5bqEcqGHsislcqWGJbWydaqhaaWcbaaabaGaemyEaKhaaaGcbaGaemOCaihaaaGaay5waiaaw2faamaaDaaaleaaaeaacqaIYaGmaaaakiaawUhacaGL9baaaeaacqaIYaGmaaaacaGLOaGaayzkaaGaeiOla4IaaCzcaiaaxMaacqGGOaakcqaIXaqmcqaIXaqmcqGGPaqkaaa@7B17@

The parameters characterizing the spots (cksx
 MathType@MTEF@5@5@+=feaafiart1ev1aaatCvAUfKttLearuWrP9MDH5MBPbIqV92AaeXatLxBI9gBaebbnrfifHhDYfgasaacH8akY=wiFfYdH8Gipec8Eeeu0xXdbba9frFj0=OqFfea0dXdd9vqai=hGuQ8kuc9pgc9s8qqaq=dirpe0xb9q8qiLsFr0=vr0=vr0dc8meaabaqaciaacaGaaeqabaqabeGadaaakeaacqWGJbWydaqhaaWcbaGaem4AaSgabaGaem4CamNaemiEaGhaaaaa@326F@, cksy
 MathType@MTEF@5@5@+=feaafiart1ev1aaatCvAUfKttLearuWrP9MDH5MBPbIqV92AaeXatLxBI9gBaebbnrfifHhDYfgasaacH8akY=wiFfYdH8Gipec8Eeeu0xXdbba9frFj0=OqFfea0dXdd9vqai=hGuQ8kuc9pgc9s8qqaq=dirpe0xb9q8qiLsFr0=vr0=vr0dc8meaabaqaciaacaGaaeqabaqabeGadaaakeaacqWGJbWydaqhaaWcbaGaem4AaSgabaGaem4CamNaemyEaKhaaaaa@3271@, *r*^*s*^, *I*^*s *^and *R*) are user-defined. For example, the coordinates cksx
 MathType@MTEF@5@5@+=feaafiart1ev1aaatCvAUfKttLearuWrP9MDH5MBPbIqV92AaeXatLxBI9gBaebbnrfifHhDYfgasaacH8akY=wiFfYdH8Gipec8Eeeu0xXdbba9frFj0=OqFfea0dXdd9vqai=hGuQ8kuc9pgc9s8qqaq=dirpe0xb9q8qiLsFr0=vr0=vr0dc8meaabaqaciaacaGaaeqabaqabeGadaaakeaacqWGJbWydaqhaaWcbaGaem4AaSgabaGaem4CamNaemiEaGhaaaaa@326F@ and cksy
 MathType@MTEF@5@5@+=feaafiart1ev1aaatCvAUfKttLearuWrP9MDH5MBPbIqV92AaeXatLxBI9gBaebbnrfifHhDYfgasaacH8akY=wiFfYdH8Gipec8Eeeu0xXdbba9frFj0=OqFfea0dXdd9vqai=hGuQ8kuc9pgc9s8qqaq=dirpe0xb9q8qiLsFr0=vr0=vr0dc8meaabaqaciaacaGaaeqabaqabeGadaaakeaacqWGJbWydaqhaaWcbaGaem4AaSgabaGaem4CamNaemyEaKhaaaaa@3271@, the radius *r*^*s *^and the ranges for *x *and *y *for each spot are defined from a user-defined array design. The user should also specify the number of dust clusters *M *on the array. The other parameters characterizing the dust are random variables, and the probability laws for their generation is a matter of choice. We use uniform distributions for *r*^*d *^(in the interval 0 to *r*_*m*_) and *I*^*d *^(in the interval 0 to *I*_*m*_), where *r*_*m *_and *I*_*m *_are a user-defined maximal dust cluster radius and maximal dust intensity, respectively. We also assume that ckdx
 MathType@MTEF@5@5@+=feaafiart1ev1aaatCvAUfKttLearuWrP9MDH5MBPbIqV92AaeXatLxBI9gBaebbnrfifHhDYfgasaacH8akY=wiFfYdH8Gipec8Eeeu0xXdbba9frFj0=OqFfea0dXdd9vqai=hGuQ8kuc9pgc9s8qqaq=dirpe0xb9q8qiLsFr0=vr0=vr0dc8meaabaqaciaacaGaaeqabaqabeGadaaakeaacqWGJbWydaqhaaWcbaGaem4AaSgabaGaemizaqMaemiEaGhaaaaa@3251@ and ckdy
 MathType@MTEF@5@5@+=feaafiart1ev1aaatCvAUfKttLearuWrP9MDH5MBPbIqV92AaeXatLxBI9gBaebbnrfifHhDYfgasaacH8akY=wiFfYdH8Gipec8Eeeu0xXdbba9frFj0=OqFfea0dXdd9vqai=hGuQ8kuc9pgc9s8qqaq=dirpe0xb9q8qiLsFr0=vr0=vr0dc8meaabaqaciaacaGaaeqabaqabeGadaaakeaacqWGJbWydaqhaaWcbaGaem4AaSgabaGaemizaqMaemyEaKhaaaaa@3253@ are uniformly distributed over the array. Statistical laws of the dust characteristics can generally be different in the two (Cy3, Cy5) channels.

In the developed simulation model we also account for the nonspecific hybridization and statistical noise:

F˜i(x,y)=Fi(x,y)+B¯i+ηBiB¯iGB+σ(x,y)GS,     (12)
 MathType@MTEF@5@5@+=feaafiart1ev1aaatCvAUfKttLearuWrP9MDH5MBPbIqV92AaeXatLxBI9gBaebbnrfifHhDYfgasaacH8akY=wiFfYdH8Gipec8Eeeu0xXdbba9frFj0=OqFfea0dXdd9vqai=hGuQ8kuc9pgc9s8qqaq=dirpe0xb9q8qiLsFr0=vr0=vr0dc8meaabaqaciaacaGaaeqabaqabeGadaaakeaacuWGgbGrgaacamaaDaaaleaacqWGPbqAaeaaaaGccqGGOaakcqWG4baEcqGGSaalcqWG5bqEcqGGPaqkcqGH9aqpcqWGgbGrdaqhaaWcbaGaemyAaKgabaaaaOGaeiikaGIaemiEaGNaeiilaWIaemyEaKNaeiykaKIaey4kaSIafmOqaiKbaebadaqhaaWcbaGaemyAaKgabaaaaOGaey4kaSIaeq4TdG2aa0baaSqaaiabdkeacjabdMgaPbqaaaaakiqbdkeaczaaraWaa0baaSqaaiabdMgaPbqaaaaakiabdEeahnaaBaaaleaacqWGcbGqaeqaaOGaey4kaSIaeq4WdmNaeiikaGIaemiEaGNaeiilaWIaemyEaKNaeiykaKIaem4raC0aaSbaaSqaaiabdofatbqabaGccqGGSaalcaWLjaGaaCzcaiabcIcaOiabigdaXiabikdaYiabcMcaPaaa@5C51@

where *i *represents either Cy3 or Cy5, B¯i
 MathType@MTEF@5@5@+=feaafiart1ev1aaatCvAUfKttLearuWrP9MDH5MBPbIqV92AaeXatLxBI9gBaebbnrfifHhDYfgasaacH8akY=wiFfYdH8Gipec8Eeeu0xXdbba9frFj0=OqFfea0dXdd9vqai=hGuQ8kuc9pgc9s8qqaq=dirpe0xb9q8qiLsFr0=vr0=vr0dc8meaabaqaciaacaGaaeqabaqabeGadaaakeaacuWGcbGqgaqeamaaDaaaleaacqWGPbqAaeaaaaaaaa@2F59@ and η_*Bi *_are the user-defined average and noise-to-signal ratio of nonspecific fluorescence intensity in the color channel *i*, σ(*x*,*y*) is the standard deviation of the pixel statistical noise, and *G*_*B *_and *G*_*S *_are independent Gaussian random variables with zero mean and unit standard deviation. The exact representation for σ(*x*,*y*) is defined by the experimental set-up. There are currently three possibilities: σ(*x*,*y*) can be (i) constant, (ii) proportional to the signal, or (iii) proportional to the square root of signal. The type and quantitative characteristics of the statistical noise are defined by the user.

## Discussion

### Software

The developed algorithm for quality control is included in the software package MAIA, which offers a complete solution for DNA microarray image analysis, including automatic spot localization and spot quantification procedures. A demo version of the software can be downloaded from [8].

### Artificial images

**Figure 6:**
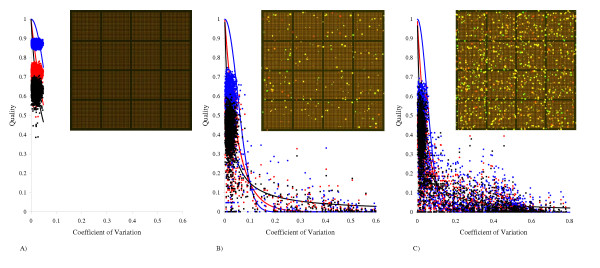
**Quality plots (*Q*_*k *_versus *V*_*k*_) for artificial images. **Three generated images differed in the percentage of dust clusters with respect to the number of good spots: A) 0%, B) 5% and C) 25%. For the further details see the text. The solid lines represent the user-defined ideal quality curves *f*(*V*_*k*_): *Red *– exponential (Eq. (6),a); *Blue *– Gaussian-like (Eq. (6),b); *Black *– inverse (Eq. (6),c).

All artificial images were generated using the same array design: 4 × 4 blocks and 21 × 21 spots within each block with the inter-spot distance of 15 pixels. For all spots in the generated arrays the spot radius, *r*, was about 4 pixels, the intensity, *I*, in the Cy3 color channel was 5000 and the ratio, *R*, of the Cy5 and Cy3 channels was 3. Non-specific hybridization was generated using B¯i
 MathType@MTEF@5@5@+=feaafiart1ev1aaatCvAUfKttLearuWrP9MDH5MBPbIqV92AaeXatLxBI9gBaebbnrfifHhDYfgasaacH8akY=wiFfYdH8Gipec8Eeeu0xXdbba9frFj0=OqFfea0dXdd9vqai=hGuQ8kuc9pgc9s8qqaq=dirpe0xb9q8qiLsFr0=vr0=vr0dc8meaabaqaciaacaGaaeqabaqabeGadaaakeaacuWGcbGqgaqeamaaDaaaleaacqWGPbqAaeaaaaaaaa@2F59@ = 1000 and η_*Bi *_= 0.5. The standard deviation of the statistical noise, σ(*x*,*y*), at each pixel was proportional to the signal at the corresponding pixel with the noise-to-signal ratio of 0.1. The three generated images (Fig. 6 (insets)) differed in the number of dust clusters. The percentages of dust clusters with respect to the number of good spots were: 0% (image 6A), 5% (image 6B) and 25% (image 6C). In all cases the maximal dust cluster radius, *r*_*m*_, was set to 8, and maximal dust intensity, *I*_*m*_, to 64000.

As all spots have the same theoretical ratio (*R *= 3), they can be considered as replicates and therefore we can use the quantitative characteristics defined in Eqs. (7) and (8) to characterize the performance of the algorithm. We expect the best estimators to provide the averaged Cy5/Cy3 ratio closer to the true ratio (*R *= 3) with the least spread around this value.

For each artificial array we compared the weighted statistical characteristics for the average R¯
 MathType@MTEF@5@5@+=feaafiart1ev1aaatCvAUfKttLearuWrP9MDH5MBPbIqV92AaeXatLxBI9gBaebbnrfifHhDYfgasaacH8akY=wiFfYdH8Gipec8Eeeu0xXdbba9frFj0=OqFfea0dXdd9vqai=hGuQ8kuc9pgc9s8qqaq=dirpe0xb9q8qiLsFr0=vr0=vr0dc8meaabaqaciaacaGaaeqabaqabeGadaaakeaacuWGsbGugaqeaaaa@2DF1@ (Eq. (7)) and for the ratio variation coefficient *V *(Eq. (8)) with the un-weighted ones. The un-weighted characteristics were obtained from Eqs. (7) and (8) by setting all *Q*_*l*_, *l *= 1,...,*n *to 1. The weighted characteristics were calculated with the overall quality values *Q*_*l *_available from the quality analysis algorithm. As all spots from the simulated image can be considered as replicates, we artificially split up the total number of spots into the groups of three closely placed spots. These groups, regarded as independent triplicates, can be used to calculate the experimental quality values *Q*_*k *_(Eq. (4)) and to build up the corresponding quality plot (*Q*_*k *_*versus V*_*k*_). Three functional shapes (Eq. (6)) for the ideal quality curve *f*(*V*_*k*_) were tested.

The results are collected in Table 1. The weighted statistical characteristics with the developed quality control eliminated bias in the average Cy5/Cy3 ratio estimates and reduced the ratio variation coefficient. Note that for "ideal" image 6A (no dust clusters) the applied quality measures retained the estimates unchanged.

**Table 1:** The average R¯
 MathType@MTEF@5@5@+=feaafiart1ev1aaatCvAUfKttLearuWrP9MDH5MBPbIqV92AaeXatLxBI9gBaebbnrfifHhDYfgasaacH8akY=wiFfYdH8Gipec8Eeeu0xXdbba9frFj0=OqFfea0dXdd9vqai=hGuQ8kuc9pgc9s8qqaq=dirpe0xb9q8qiLsFr0=vr0=vr0dc8meaabaqaciaacaGaaeqabaqabeGadaaakeaacuWGsbGugaqeaaaa@2DF1@ (Eq. (7)) and the coefficient of variation *V *(Eq. (8)) of the Cy5/Cy3 ratios over all spots for three artificial images A, B, and C from Fig 6

Image	A		B		C	
	R¯ MathType@MTEF@5@5@+=feaafiart1ev1aaatCvAUfKttLearuWrP9MDH5MBPbIqV92AaeXatLxBI9gBaebbnrfifHhDYfgasaacH8akY=wiFfYdH8Gipec8Eeeu0xXdbba9frFj0=OqFfea0dXdd9vqai=hGuQ8kuc9pgc9s8qqaq=dirpe0xb9q8qiLsFr0=vr0=vr0dc8meaabaqaciaacaGaaeqabaqabeGadaaakeaacuWGsbGugaqeaaaa@2DF1@	*V*	R¯ MathType@MTEF@5@5@+=feaafiart1ev1aaatCvAUfKttLearuWrP9MDH5MBPbIqV92AaeXatLxBI9gBaebbnrfifHhDYfgasaacH8akY=wiFfYdH8Gipec8Eeeu0xXdbba9frFj0=OqFfea0dXdd9vqai=hGuQ8kuc9pgc9s8qqaq=dirpe0xb9q8qiLsFr0=vr0=vr0dc8meaabaqaciaacaGaaeqabaqabeGadaaakeaacuWGsbGugaqeaaaa@2DF1@	*V*	R¯ MathType@MTEF@5@5@+=feaafiart1ev1aaatCvAUfKttLearuWrP9MDH5MBPbIqV92AaeXatLxBI9gBaebbnrfifHhDYfgasaacH8akY=wiFfYdH8Gipec8Eeeu0xXdbba9frFj0=OqFfea0dXdd9vqai=hGuQ8kuc9pgc9s8qqaq=dirpe0xb9q8qiLsFr0=vr0=vr0dc8meaabaqaciaacaGaaeqabaqabeGadaaakeaacuWGsbGugaqeaaaa@2DF1@	*V*

No quality control	3.00	0.028	2.96	0.20	2.84	0.35
*f*(*V*_*k *_) =exp{-*V*_*k*_/V¯ MathType@MTEF@5@5@+=feaafiart1ev1aaatCvAUfKttLearuWrP9MDH5MBPbIqV92AaeXatLxBI9gBaebbnrfifHhDYfgasaacH8akY=wiFfYdH8Gipec8Eeeu0xXdbba9frFj0=OqFfea0dXdd9vqai=hGuQ8kuc9pgc9s8qqaq=dirpe0xb9q8qiLsFr0=vr0=vr0dc8meaabaqaciaacaGaaeqabaqabeGadaaakeaacuWGwbGvgaqeaaaa@2DF9@}	3.00	0.028	2.99	0.041	2.99	0.089
*f*(*V*_*k *_) =exp{-Vk2 MathType@MTEF@5@5@+=feaafiart1ev1aaatCvAUfKttLearuWrP9MDH5MBPbIqV92AaeXatLxBI9gBaebbnrfifHhDYfgasaacH8akY=wiFfYdH8Gipec8Eeeu0xXdbba9frFj0=OqFfea0dXdd9vqai=hGuQ8kuc9pgc9s8qqaq=dirpe0xb9q8qiLsFr0=vr0=vr0dc8meaabaqaciaacaGaaeqabaqabeGadaaakeaacqWGwbGvdaqhaaWcbaGaem4AaSgabaGaeGOmaidaaaaa@305F@/V¯ MathType@MTEF@5@5@+=feaafiart1ev1aaatCvAUfKttLearuWrP9MDH5MBPbIqV92AaeXatLxBI9gBaebbnrfifHhDYfgasaacH8akY=wiFfYdH8Gipec8Eeeu0xXdbba9frFj0=OqFfea0dXdd9vqai=hGuQ8kuc9pgc9s8qqaq=dirpe0xb9q8qiLsFr0=vr0=vr0dc8meaabaqaciaacaGaaeqabaqabeGadaaakeaacuWGwbGvgaqeaaaa@2DF9@^2^}	3.00	0.028	2.99	0.038	2.99	0.097
*f*(*V*_*k *_) =1/{1 + *V*_*k*_/V¯ MathType@MTEF@5@5@+=feaafiart1ev1aaatCvAUfKttLearuWrP9MDH5MBPbIqV92AaeXatLxBI9gBaebbnrfifHhDYfgasaacH8akY=wiFfYdH8Gipec8Eeeu0xXdbba9frFj0=OqFfea0dXdd9vqai=hGuQ8kuc9pgc9s8qqaq=dirpe0xb9q8qiLsFr0=vr0=vr0dc8meaabaqaciaacaGaaeqabaqabeGadaaakeaacuWGwbGvgaqeaaaa@2DF9@}	3.00	0.028	2.99	0.042	2.99	0.094

The three compared ideal quality shapes *f*(*V*_*k*_) (Eq. (6)) demonstrated similar performance. A small advantage (slightly smaller *V*) of the Gaussian-like function (Eq. (6),b) for image 6B (5% of dust clusters) was compensated by the lowest performance for image 6C (25% of dust clusters). The difference between the three shapes can be seen from Fig 6, where the corresponding quality plots are drawn for the three generated images. The inverse shape (Eq. (6),c) is the least stringent whereas the Gaussian-like shape is the most stringent with respect to the replicates variability. This means that the inverse shape does not require the replicates with higher variability to have lower quality values (and correspondingly the replicates with smaller variability to have higher quality values) as strictly as the Gaussian-like one. Although it seems that the Gaussian-like shape is the best choice, there is still a drawback. Due to its relatively abrupt decrease it is difficult to keep the balance in fitting weights ψ_*k *_between the head and the tail of the shape. Despite the adaptive selection of the fitting weights ψ_*k *_(see section Spot quality analysis), the fitting procedure, trying to ensure the highest quality for the replicates with the lowest variability, may still overlook the replicates with the higher variability. This depends on the image quality (replicate variability) and can explain why the Gaussian-like shape yielded the greatest ratio variation for image 6C. This also indicates that more work is needed to find out an optimal combination of the ideal quality curve and the fitting weights ψ_*k *_to be used in the training procedure.

The results of simulation study and our experience with the experimental images suggest that the exponential shape (Eq. (6),a) offers a reasonable compromise between the fitting weights used and stringency with respect to the replicates variability. Therefore in the further quality analysis we will apply exponential *f*(*V*_*k*_).

### Experimental images

We performed quality analysis of two experimental images with different array designs and signal-to-noise levels.

**Figure 7:**
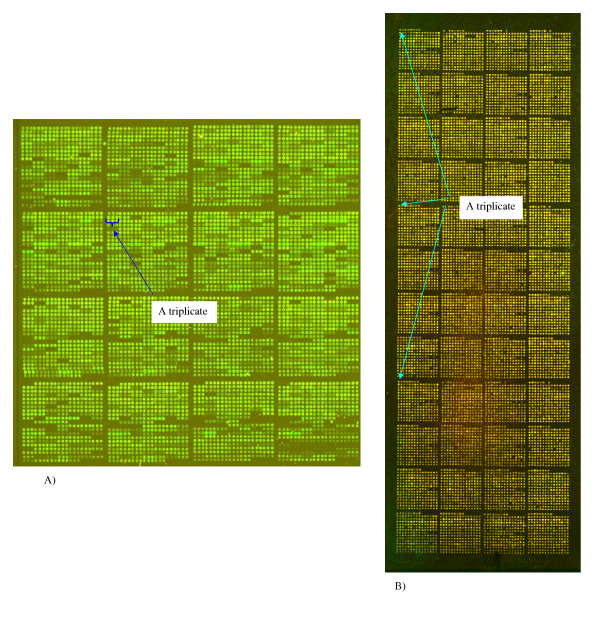
**Experimental images. **A) 4 × 4 blocks with 21 × 21 spots per block, spot cell size is about 10 pixels; B) 12 × 4 blocks with 15 × 15 spots per block, spot cell size is about 30 pixels. The locations of triplicates are indicated.

One image (Fig. 7A) (image 7A) was provided as a demonstration example for UCSF Spot 2.0 (downloadable from [16]). It contains 4 × 4 blocks with 21 × 21 spots in each block, with a spot cell size of about 10 pixels. Cy3 and Cy5 color channels are strongly correlated, with the average correlation coefficient for the spots being about 0.97. Bright contamination spots can be seen irregularly scattered over the array. The magnified image of one such spot is shown in Fig. 2 (inset). Each clone was spotted in triplicate. The replicated spots are placed as neighbors in a row (see Fig. 7A).

The second image (from the Institute Curie) (image 7B) contains 12 × 4 blocks with 12 × 12 spots in each block (Fig. 7B), with a spot cell size of about 30 pixels. The average correlation between the channels for the spots is about 0.85. This is lower than for the first image, although this image has no obvious contamination spots. Each clone was prepared in triplicate, with the replicated spots in three vertically distributed sub-arrays (see Fig. 7B).

We expect the Cy5/Cy3 ratios from the replicates to be similar. Therefore it is reasonable to take the coefficient of variation (Eq. (8)) of the replicates as a quantitative measure of the ratio estimation consistency. However, this measure may not be totally objective: (i) the estimates may be consistent, but systematically biased (the true values of the ratios are unknown); (ii) three replicated spots of very poor quality may give very similar ratio values just by chance (the number of replicates is low).

The average over all replicates at the given array coefficient of variation is taken as a global indicator of the Cy5/Cy3 ratio consistency of the array.

We compared two quality characteristics (the coefficient of determination *CD *(*q*_*1*_) and the coefficient of variation of two ratio estimates *CVR *(*q*_*7*_), applied separately) and the overall quality parameter *Q *(Eq. (1)) to the case when no quality control was applied. The weights of the marginal quality parameters for *Q *were identified using the exponential ideal quality curve (Eq. (6),a) with V¯
 MathType@MTEF@5@5@+=feaafiart1ev1aaatCvAUfKttLearuWrP9MDH5MBPbIqV92AaeXatLxBI9gBaebbnrfifHhDYfgasaacH8akY=wiFfYdH8Gipec8Eeeu0xXdbba9frFj0=OqFfea0dXdd9vqai=hGuQ8kuc9pgc9s8qqaq=dirpe0xb9q8qiLsFr0=vr0=vr0dc8meaabaqaciaacaGaaeqabaqabeGadaaakeaacuWGwbGvgaqeaaaa@2DF9@ ≈ 0.08 for image 7A, and with V¯
 MathType@MTEF@5@5@+=feaafiart1ev1aaatCvAUfKttLearuWrP9MDH5MBPbIqV92AaeXatLxBI9gBaebbnrfifHhDYfgasaacH8akY=wiFfYdH8Gipec8Eeeu0xXdbba9frFj0=OqFfea0dXdd9vqai=hGuQ8kuc9pgc9s8qqaq=dirpe0xb9q8qiLsFr0=vr0=vr0dc8meaabaqaciaacaGaaeqabaqabeGadaaakeaacuWGwbGvgaqeaaaa@2DF9@ ≈ 0.2 for image 7B. The results are summarized in Table 2.

**Table 2:** The average over all replicates coefficients of variation *V *(Eq. (8)) for two experimental images A and B from Fig. 7

Quality Control	A	B
No control	0.066	0.120
*CD*	0.019	0.105
*CVR*	0.028	0.102
Overall *Q*	0.017	0.101

We found a greater improvement for image 7A than for image 7B after applying the quality measures. This was not a surprise, as image 7B is characterized by a reasonably high signal-to-noise level, and it does not contain any obvious contaminated spots. However, even in this case the quality measures cannot be ignored, as there are still a few low-intensity spots that need to be specially treated (probably rejected). By contrast, image 7A has obvious randomly distributed pieces of dust, and the developed quality measures proved to be powerful enough to disregard the contaminated spots, thus increasing the consistency of the Cy5/Cy3 ratio estimates.

The comparison of the different quality measures has shown that they are array-specific. For image 7B, *CD*, *CVR *and *Q *gave almost equivalent performance, whereas for image 7A, *CD *was much better than *CVR*, and *Q *gave a slightly better ratio consistency than both of these. However, the gain from *Q *was not much greater, so that it would be reasonable to assume that only a single quality measure (such as *CD *or *CVR*) could always give good results. Unfortunately, this is not the case. Although we have found that *CD *and *CVR *are indeed the most powerful quality measures they cannot cover every type of distortion.

We demonstrate this with image 7A. There remained, after applying the individual quality measures, several triplicates with high coefficients of variation that were not correctly suppressed. Three such replicates are shown in Fig. 5, and the main quantitative characteristics of the corresponding spots are listed in Table 3. Note that Table 3 contains the quality characteristics from section Quality characteristics, which do not need to reside in the interval [0;1].

**Table 3:** The main characteristics of the spots from three triplicates (see Fig. 5 (insets)), demonstrating excessive variability in the Cy5/Cy3 ratio estimates

Triplicate	Quality Parameter	Spot 1	Spot 2	Spot 3
A	Ratio	0.27	0.26	0.12
	*CD*	0.98	0.98	0.97
	*CVR*	0.016	0.014	0.11
	*IS*	0.17	0.21	1.02
	*UB*	0.060	0.10	0.31

B	Ratio	0.19	0.13	0.072
	*CD*	0.85	0.60	0.33
	*CVR*	0.010	0.014	0.012
	*IS*	0.15	0.072	0.041
	*UB*	0.041	0.030	0.042

C	Ratio	0.29	0.30	0.44
	*CD*	0.97	0.97	0.93
	*CVR*	0.010	0.011	0.0052
	*IS*	0.12	0.15	0.36
	*UB*	0.24	0.11	0.99

In triplicate A, one spot is clearly contaminated; however, this contamination is highly correlated in the two channels (*CD *= 0.97) and the spots cannot be filtered out using *CD*. A more powerful parameter for this type of problem is *CVR *(0.11), perhaps in combination with *IS *(1.02). Triplicate B includes low-intensity spots. Although *CVR *quality characteristic does not detect any deficiency in this case, the difference between the estimated intensity ratios (within the triplicate) is large, meaning that *CVR *alone is not sufficient to eliminate the outlier spots. *CD *seems to be more indicative in this case. Triplicate C cannot be confidently recognized either by *CD *or *CVR *quality measures. This problem (contamination penetrating into the spot from the outside area) can be figured out by applying either the uniformity of the background quality parameter (*UB *= 0.99) or the Durbin-Watson quality parameter (*DWS *= 0.58).

This type of analysis leads to the refined critical values xilim⁡
 MathType@MTEF@5@5@+=feaafiart1ev1aaatCvAUfKttLearuWrP9MDH5MBPbIqV92AaeXatLxBI9gBaebbnrfifHhDYfgasaacH8akY=wiFfYdH8Gipec8Eeeu0xXdbba9frFj0=OqFfea0dXdd9vqai=hGuQ8kuc9pgc9s8qqaq=dirpe0xb9q8qiLsFr0=vr0=vr0dc8meaabaqaciaacaGaaeqabaqabeGadaaakeaacqWG4baEdaqhaaWcbaGaemyAaKgajeaybaGagiiBaWMaeiyAaKMaeiyBa0gaaaaa@3435@ for each of the quality characteristics. These xilim⁡
 MathType@MTEF@5@5@+=feaafiart1ev1aaatCvAUfKttLearuWrP9MDH5MBPbIqV92AaeXatLxBI9gBaebbnrfifHhDYfgasaacH8akY=wiFfYdH8Gipec8Eeeu0xXdbba9frFj0=OqFfea0dXdd9vqai=hGuQ8kuc9pgc9s8qqaq=dirpe0xb9q8qiLsFr0=vr0=vr0dc8meaabaqaciaacaGaaeqabaqabeGadaaakeaacqWG4baEdaqhaaWcbaGaemyAaKgajeaybaGagiiBaWMaeiyAaKMaeiyBa0gaaaaa@3435@ values are then recalculated into the corresponding weights *w*_*i *_for the user-selected overall quality threshold *Q*^lim^, using Eq. (2) (see Fig. 4). In general, however, the weights are derived automatically from Eq. (5) by the non-linear fitting procedure. If certain quality factors do not influence the shape of the experimental quality curve *Q*_*E *_(Eq. (4)), the corresponding weights will be set close to 0. If a certain effect shows up in only a small number of spots, it may be neglected by the optimization procedure, and the corresponding weight will be erroneously small. In this case, manual correction of the weights would be necessary.

The quality value of three bad replicates from Fig. 5 (insets), as well as of many others demonstrating larger deviations in the obtained Cy5/Cy3 ratio estimates (Fig. 5), were decreased using the combined criteria *Q*, whereas the quality of replicates with smaller variations remained almost unaffected.

Quality measures can signal some of the replicates to be bad despite there being no big difference in the ratios. With a small number of replicated spots all spots from a replicate may indeed have very close ratios, but several of them may be really deficient. Therefore, it is more important to consider those replicates demonstrating unusually high diversity in the obtained ratio estimates. This clearly suggests problems in the spots. Algorithmically, this can be achieved by assigning larger weights ψ_*k *_to these replicates in the non-linear fitting procedure (Eq. (5)). The fitted parameters *w*_*i *_are then used to flag out all deficient spots, even those that belong to the replicates with the consistent ratio estimates. However, if *all *spots from a consistent replicate demonstrate poor quality, this can also be an indication that this replicate is actually good, but it was erroneously flagged out by the quality analysis procedure. This would require user intervention to correct for the selected set of quality characteristics and/or for the corresponding quality weight *w*_*i*_. Additional procedures for automatic analysis of the replicates with consistent ratios but low quality values can be envisaged.

The fact that overall *Q *does not show up much better performance (Table 2) is due to rather good general quality of the images, and a few problematic triplicates cannot influence very much the averaged coefficients of variation. For example, in image 7A, we have less than 9% of triplicates with the ratio variation coefficients larger that the selected V¯
 MathType@MTEF@5@5@+=feaafiart1ev1aaatCvAUfKttLearuWrP9MDH5MBPbIqV92AaeXatLxBI9gBaebbnrfifHhDYfgasaacH8akY=wiFfYdH8Gipec8Eeeu0xXdbba9frFj0=OqFfea0dXdd9vqai=hGuQ8kuc9pgc9s8qqaq=dirpe0xb9q8qiLsFr0=vr0=vr0dc8meaabaqaciaacaGaaeqabaqabeGadaaakeaacuWGwbGvgaqeaaaa@2DF9@ (~0.08), and 7% for image 7B (V¯
 MathType@MTEF@5@5@+=feaafiart1ev1aaatCvAUfKttLearuWrP9MDH5MBPbIqV92AaeXatLxBI9gBaebbnrfifHhDYfgasaacH8akY=wiFfYdH8Gipec8Eeeu0xXdbba9frFj0=OqFfea0dXdd9vqai=hGuQ8kuc9pgc9s8qqaq=dirpe0xb9q8qiLsFr0=vr0=vr0dc8meaabaqaciaacaGaaeqabaqabeGadaaakeaacuWGwbGvgaqeaaaa@2DF9@ ≈ 0.2).

### Replicated arrays

**Figure 8:**
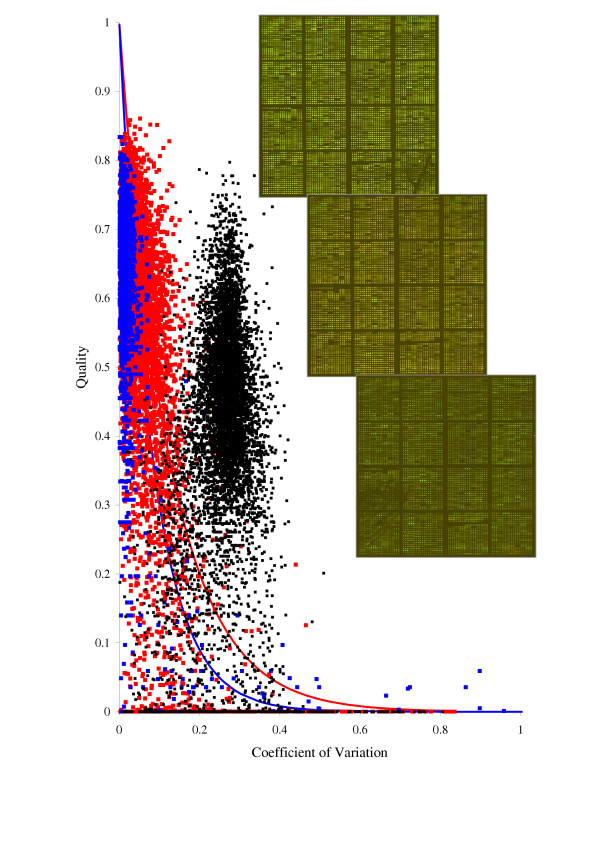
**Quality plots (*Q*_*k *_versus *V*_*k*_) using replicated arrays. ***Black dots *– three replicated images (insets) are combined without normalization and the default quality weights are used; *red dots *– three replicated images (insets) are combined after the global normalization; *blue dots *– triplicate spots from the first array are used. The solid lines represent the exponential ideal quality curves *f*(*V*_*k*_) (Eq. (6),a): *Red line *(for the red dots) – V¯
 MathType@MTEF@5@5@+=feaafiart1ev1aaatCvAUfKttLearuWrP9MDH5MBPbIqV92AaeXatLxBI9gBaebbnrfifHhDYfgasaacH8akY=wiFfYdH8Gipec8Eeeu0xXdbba9frFj0=OqFfea0dXdd9vqai=hGuQ8kuc9pgc9s8qqaq=dirpe0xb9q8qiLsFr0=vr0=vr0dc8meaabaqaciaacaGaaeqabaqabeGadaaakeaacuWGwbGvgaqeaaaa@2DF9@ ≈ 0.125; Blue line (for the blue dots) – V¯
 MathType@MTEF@5@5@+=feaafiart1ev1aaatCvAUfKttLearuWrP9MDH5MBPbIqV92AaeXatLxBI9gBaebbnrfifHhDYfgasaacH8akY=wiFfYdH8Gipec8Eeeu0xXdbba9frFj0=OqFfea0dXdd9vqai=hGuQ8kuc9pgc9s8qqaq=dirpe0xb9q8qiLsFr0=vr0=vr0dc8meaabaqaciaacaGaaeqabaqabeGadaaakeaacuWGwbGvgaqeaaaa@2DF9@ ≈ 0.08.

Quality analysis can also be performed with replicated spots from different arrays. Three replicated images (Fig. 8 (insets)) were used for quality analysis. Although each image contains three replicated spots placed as neighbors in a row (similar to image A from Fig. 7), we pretended that there were no replicates within each array. Therefore we made available only replicated spots from different arrays for quality analysis. In Fig. 8 we present three quality plots. Black dots correspond to the case when the three replicated images were combined without normalization and the default quality weights were used. Relatively large coefficients of variation are due to the bias in the obtained Cy5/Cy3 ratio estimates between the three images. To apply our algorithm of quality analysis in this case the ideal quality curve should account for this bias, i.e. it should implicitly incorporate image normalization model. A simpler way would be to separate the normalization and quality analysis procedures: the image normalization should precede the quality analysis, or we can also envisage an iterative procedure where the steps of normalization and quality analysis are performed in turn. Taking into account that a variety of normalization methods [17] are currently available, we leave the detailed development of the quality analysis strategy in this case for the future.

As an example, we applied a global normalization algorithm [18]. It is assumed that most genes are not differentially expressed and therefore the expected averaged over all spots of the array Cy5/Cy3 ratio should be close to one. The normalization constant *a *for each array can be calculated as a=exp⁡{∑i=1nln⁡Ri/n}
 MathType@MTEF@5@5@+=feaafiart1ev1aaatCvAUfKttLearuWrP9MDH5MBPbIqV92AaeXatLxBI9gBaebbnrfifHhDYfgasaacH8akY=wiFfYdH8Gipec8Eeeu0xXdbba9frFj0=OqFfea0dXdd9vqai=hGuQ8kuc9pgc9s8qqaq=dirpe0xb9q8qiLsFr0=vr0=vr0dc8meaabaqaciaacaGaaeqabaqabeGadaaakeaacqWGHbqycqGH9aqpcyGGLbqzcqGG4baEcqGGWbaCdaGadaqaamaalyaabaWaaabCaeaacyGGSbaBcqGGUbGBcqWGsbGudaWgaaWcbaGaemyAaKgabeaaaeaacqWGPbqAcqGH9aqpcqaIXaqmaeaacqWGUbGBa0GaeyyeIuoaaOqaaiabd6gaUbaaaiaawUhacaGL9baaaaa@434E@, where *n *is the number of spots and *R*_*i *_is the ratio estimate for the *i*-th spot. Red dots in Fig 8 represent the quality curve using the replicated arrays after the global normalization. For comparison, blue dots show the quality curve using the triplicates from the first array. The red-dot cloud spreads wider, because, even after normalization, the variability of replicates from different arrays is higher than from the same array (where replicates were closely placed). Despite this replicated arrays can be used in the quality analysis since the required tendency – the decrease of the overall quality with the increase of the replicate variability – can be ensured. To account for higher levels of inter-array statistical variability in replicates, we have to apply less stringent ideal quality curve. This can be achieved by selecting larger values for the parameter V¯
 MathType@MTEF@5@5@+=feaafiart1ev1aaatCvAUfKttLearuWrP9MDH5MBPbIqV92AaeXatLxBI9gBaebbnrfifHhDYfgasaacH8akY=wiFfYdH8Gipec8Eeeu0xXdbba9frFj0=OqFfea0dXdd9vqai=hGuQ8kuc9pgc9s8qqaq=dirpe0xb9q8qiLsFr0=vr0=vr0dc8meaabaqaciaacaGaaeqabaqabeGadaaakeaacuWGwbGvgaqeaaaa@2DF9@ in Eq. (6).

### Quality weight extrapolation

**Figure 9:**
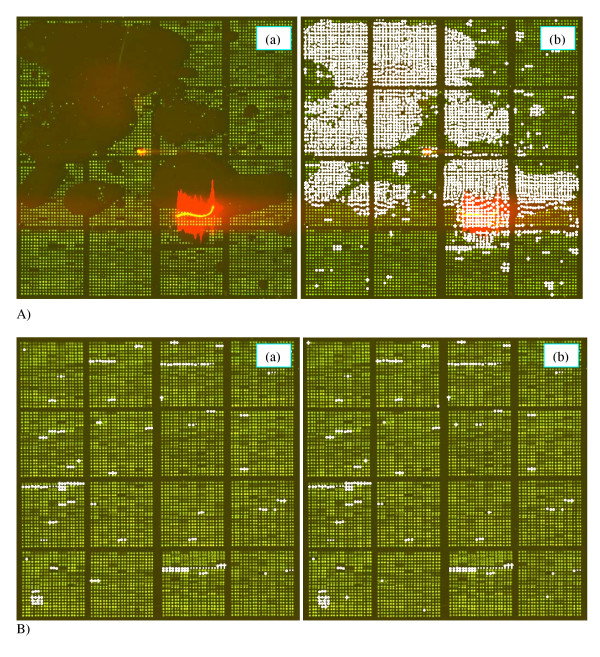
**Quality analysis of poor-quality (A) and good-quality (B) images. **A: (a) Poor-quality image; (b) Image A with the "bad" spots identified by the quality analysis based on its own triplicates. B: (a) Image B with the bad spots identified by the quality analysis based on the triplicates from image A; (b) Image B with the bad spots identified by the quality analysis based on its own triplicates. "Bad" spots are the spots with the overall quality below 0.3. "Bad" spots are indicated by the white crosses.

We demonstrate here a possibility to apply quality weights obtained from the analysis of one training image, which should contain replicated spots, to other arrays, which may not contain replicates. We used two images (Figs. 9A and 9B) of the same design as before: each image contains three replicated spots placed as neighbors in a row. Image 9A has obvious deficiencies (large amount of absent spots, scratches, contamination), whereas image 9B is less problematic.

The quality weights were estimated from the triplicates of image 9A, as it contains a wider diversity of possible artifacts and distortions. Using the obtained weights the "bad" spots were identified in image 9A. A spot was classified as a bad spot if its overall quality was below 0.3. Fig. 9A(b) shows the same image with the "bad" spots indicated by the white crosses.

To eliminate the "bad" spots (*Q *< 0.3) from image 9B, we first applied the quality weights obtained for image 9A. The result is shown in Fig. 9B(a). Then we performed quality analysis for image 9B using its own triplicates. The spots flagged out by this approach are indicated in Fig. 9B(b). Comparing Figs. 9B(a) and 9B(b), one can conclude that the results are very similar, although not identical, of course.

This example attempts to reproduce an important possibility of designing microarray experiments. A small number of training arrays with replicated spots and representative diversity of possible artifacts can be measured and analyzed. The obtained results can then be used to evaluate the quality of other arrays of similar design, which may not contain replicated spots.

## Conclusion

We have described an algorithm for quantitative spot quality evaluation in DNA microarray image analysis that allows the automatic identification of the weights for the marginal quality parameters within the model combining these parameters into an overall spot quality value. The algorithm relies on the assumption that unspoiled replicated spots should have higher levels of consistency in the obtained Cy5/Cy3 ratio estimates than "bad" spots. The user is only required to define an ideal quality curve *f*(*V*_*k*_) establishing how fast the overall quality of the replicates must decrease with increasing ratio variation in the corresponding replicates. For simple models from Eq. (6) only one parameter – the characteristic ratio variation coefficient V¯
 MathType@MTEF@5@5@+=feaafiart1ev1aaatCvAUfKttLearuWrP9MDH5MBPbIqV92AaeXatLxBI9gBaebbnrfifHhDYfgasaacH8akY=wiFfYdH8Gipec8Eeeu0xXdbba9frFj0=OqFfea0dXdd9vqai=hGuQ8kuc9pgc9s8qqaq=dirpe0xb9q8qiLsFr0=vr0=vr0dc8meaabaqaciaacaGaaeqabaqabeGadaaakeaacuWGwbGvgaqeaaaa@2DF9@ – must be specified. In this paper the functional shape for *f*(*V*_*k*_) was empirically selected through numerous experiments with artificial and experimental images. This, however, may not be an optimal choice and further improvements can be expected, preferably using more theoretical approaches. A complementary perspective is to further elaborate the algorithm for estimating the fitting weights ψ_*k*_, which may be implicitly dependent on the ideal quality curve.

We use nine marginal quality characteristics, which cover a broad range of different deviations from a normal (good) spot. Therefore, it is possible that some of these parameters will not be relevant for a certain image. For example, if there are no clearly un-circular spots, the corresponding parameter (*GS*) can be omitted. The optimization procedure will however report this, assigning a very low weight to the corresponding quality characteristic. These weights are then converted into the critical levels xilim⁡
 MathType@MTEF@5@5@+=feaafiart1ev1aaatCvAUfKttLearuWrP9MDH5MBPbIqV92AaeXatLxBI9gBaebbnrfifHhDYfgasaacH8akY=wiFfYdH8Gipec8Eeeu0xXdbba9frFj0=OqFfea0dXdd9vqai=hGuQ8kuc9pgc9s8qqaq=dirpe0xb9q8qiLsFr0=vr0=vr0dc8meaabaqaciaacaGaaeqabaqabeGadaaakeaacqWG4baEdaqhaaWcbaGaemyAaKgajeaybaGagiiBaWMaeiyAaKMaeiyBa0gaaaaa@3435@ using Eq. (2), which will tolerate a big diversity in the possible values of the corresponding parameter. Thus, the developed procedure both searches for the weights and implicitly reports on the relevance of the quality parameters to each particular image. However, if the images used to train the model (that is to identify the weights) are not representative enough and do not contain enough spots with certain types of artifacts, some important sources of spot deficiency may be overlooked. In this case, manual adjustment of the weights may be necessary.

Another problem is that generally a very small number of replicated spots (rarely more than 3) are available for the analysis. Therefore it is possible that all spots from a replicate, being defective, demonstrate consistent Cy5/Cy3 ratio values. However, we expect that is less probable than to observe unusually high diversity in the ratio estimates for these replicates. If all spots from a consistent replicate are actually good, but they are erroneously assigned low quality values, this is an indication of some problems in the quality analysis itself. This situation would possibly require user participation to correct for the selected set of quality characteristics and/or for the corresponding quality weights *w*_*i*_.

We have demonstrated a possibility to carry out the quality analysis using replicated spots from different arrays. An additional procedure of the image normalization should precede the quality analysis in this case.

## Authors' contributions

EN conceived the algorithm, performed software implementation and drafted the manuscript. EB conceived of the study and participated in coordination. All authors read and approved the final manuscript.
